# Functional PET imaging of gut microbiota with [^18^F]fluorodeoxyglucose, [^18^F]fluorodeoxysorbitol and [^11^C]choline reflects *Clostridia* and *Lactobacillales* abundance in caecum and small intestine and host metabolic interactions

**DOI:** 10.1007/s00259-026-07988-y

**Published:** 2026-06-13

**Authors:** Federica La Rosa, Maria Angela Guzzardi, Gabriele Conti, Debora Petroni, Mercedes Pardo Tendero, Silvia Bernardi, Monica Barone, Daniele Panetta, Lorena Tedeschi, Costanza Fabbri, Federico Casavecchia, Daria Riabitch, Federico Granziera, Rosetta Ragusa, Chiara Caselli, Assuero Giorgetti, Daniela Campani, Emma Baglini, Luca Menichetti, Philip Elsinga, Gert Luurtsema, Patrizia Brigidi, Patricia Iozzo

**Affiliations:** 1 Institute of Clinical Physiology, National Research Council (CNR), via Moruzzi 1, Pisa, 56124 Italy; 2 Human Microbiomics Unit, Department of Medical and Surgical Sciences, University of Bologna, via Massarenti 9, Bologna, 40138 Italy; 3 Department of Pathology, University of Valencia/Health Research Institute Incliva, Blasco ibañez 15, Valencia, 46010 Spain; 4 Scuola Superiore di Studi Universitari Sant’Anna, Piazza Martiri della Libertà, Pisa, Italy; 5 Department of Chemistry, Biotechnologies and Pharmacy, University of Siena, Siena, Italy; 6 Fondazione Toscana Gabriele Monasterio, via Moruzzi 1, Pisa, 56124 Italy; 7 Department of Surgical, Medical, Molecular Pathology and Critical Care Medicine, Division of Pathology, Pisa University Hospital, Pisa, 56124 Italy; 8 Department of Nuclear Medicine and Molecular Imaging, University Medical Center Groningen, University of Groningen, Hanzeplein 1, 9713GZ, Groningen, The Netherlands; 9 APC Microbiome Ireland, University College Cork, Cork, Ireland

**Keywords:** PET, Microbiota, Glucose, Choline, Sorbitol, Chronic disease

## Abstract

**Purpose:**

Interaction of gut microbiota (GM) with dietary sugars (glucose, sorbitol) and choline has been transversely implicated in the pathogenesis of multiple chronic diseases. Our aim was to develop functional PET imaging of GM, using a multi-tracer approach to capture bacteria classes involved in sugar fermentation and choline catabolism at their gastrointestinal (GI) location.

**Methods:**

Adult and young sex-balanced groups of mice underwent oral administration of [^18^F]FDG, [^18^F]FDS or [^11^C]choline ([^11^C]cho) and repeated PET imaging over 4–5 h. Antibiotics, probiotic or faecal microbiota transplantation (FMT) served to quantify the specific role and site of bacteria action. GM was sequenced ex-vivo; gut histology and metabolic profiles were assessed in subsets.

**Results:**

[^18^F]FDG and [^18^F]FDS reflected caecum abundance of *Clostridia* and *Bacteroidia* fermenters, with [^18^F]FDG exhibiting strongest and broadest relations. Clearance of [^11^C]cho from small gut reflected *Bacilli* and *Lactobacilli* abundance. In vitro cultures supported these relationships. Urinary ^11^C-excretion was nearly abolished by antibiotics. PET imaging was able to differentiate and predict gut bacteria classes in mice receiving FMT from two age-extreme human donors. Urinary [^18^F]FDS excretion reflected small-gut goblet cell activation; high caecum [^18^F]FDG retention and small gut [^11^C]cho clearance predicted body glucose use and low systemic inflammation.

**Conclusion:**

Imaging of ingested probes is simple and effective to map GM characteristics in situ and the functional crosstalk with host processes in mice in real-time. Our data confirm that the GI ecosystem is highly diversified, pointing to small intestine and caecum GM as dominant players in gut-body handling of our target nutrients.

**Supplementary Information:**

The online version contains supplementary material available at 10.1007/s00259-026-07988-y.

## Introduction

Interaction of gut microbiota (GM) with dietary sugars (glucose, sorbitol) and choline has been implicated in the pathogenesis of multiple chronic diseases across metabolic, neurologic, cardiologic and oncologic sectors [1–12]. GM communities are spatially structured along the gastrointestinal (GI) tract, reflecting profound differences in luminal substrates, oxygen tension, pH, transit time, and host-derived factors [13]. As a result, GM composition and metabolic activity are compartmentalized. The small intestine is primarily involved in transforming and absorbing nutrients and drugs, and in immune-endocrine signalling, affecting intersubject susceptibility to diseases and drug responses; caecum and colon serve as dominant sites of microbial fermentation. This spatial organization is critical for shaping host metabolic and inflammatory homeostasis, yet remains largely inaccessible to non-invasive functional assessment [14–16].

Faecal samples are currently the most common source of GM characterization, which does not distinguish the intestinal origin of bacteria and cannot provide any direct, real-time functional gut-to-body axes insight. Our objective is to establish functional positron emission tomography (PET) imaging of GM at its intestinal location (mapping), and its dynamic exchange with body organs, affecting fates of glucose, sorbitol and choline, and hormonal/inflammatory homeostasis.

PET is a non-invasive imaging technology, utilizing radiotracers to visualize the uptake and kinetics of labelled probes in situ over time. In vitro, ^18^F-sugar analogues, like fluorodeoxysorbitol ([^18^F]FDS) and fluorodeoxyglucose ([^18^F]FDG) have been shown to enter and be trapped into cultured bacteria with differential selectivity [17], providing complementary information. So far, [^18^F]FDS in vivo has been given intravenously to image enterobacteria infections [18], since sorbitol is not extracted by body tissues. A previous study used [^18^F]FDS orally to show that its gut lumen retention was abated by antibiotics and restored by bacteria reimplant [19]. Relations with GM were not assessed. In that study [19], the physiological distribution of [^18^F]FDG to body organs beyond GM was considered a limitation. To our objective, acquiring simultaneous information on gut bacteria uptake, gut glucose absorption, and organ glucose uptake is an added value. In fact, our previous mouse study [20] with orally administered [^18^F]FDG suggests that [^18^F]FDG retention in specific gut regions, likely attributable to bacteria, may regulate bioavailability and distribution of glucose in body organs, and hormonal responses.

This study aimed to establish functional PET imaging of GM in its native intestinal environment, leveraging the oral administration of PET probes in a multi-tracer approach to capture complementary aspects of microbial-host metabolic interactions. Notably, the tracers used in this study are not intended to label single bacteria species, but GM communities responsible for given metabolic actions; in fact, GM is characterized by high gene redundancy (i.e., phylogenetically distinct microbes can perform similar metabolic functions), and the fate of glucose, sorbitol and choline depends on a balance between communities and not on single species. Thus, [^18^F]FDG was adopted as probe for glucose-utilizing, fermenting bacterial communities, in parallel undergoing intestinal glucose absorption and systemic organ uptake, whose balance is relevant to metabolic syndrome, neurologic and oncological diseases. Oral [^18^F]FDS was employed as substrate for sorbitol-utilizing and facultatively fermenting bacterial populations that may expand under antibiotic-perturbed or dysbiosis conditions, with relevance to intestinal barrier integrity, polyol intolerance, inflammatory diseases, and research on food sweeteners. Finally, [^11^C]choline ([^11^C]cho) was used to probe bacterial communities influencing intestinal choline handling and downstream enterohepatic circulation and urinary excretion by the host, as potential new targets in e.g., liver and cardiovascular oxidative damage. GM sequencing was carried out in different GI tracts to relate PET imaging signals with relative abundance of specific bacteria taxa, followed by in vitro demonstration of tracer use by selected cultured bacteria. Gut histology and systemic glucose, hormone, and inflammation profiles were also explored in the perspective of GM imaging to serve as biomarker.

## Methods

### Study design (Fig. 1)

**Fig. 1:**
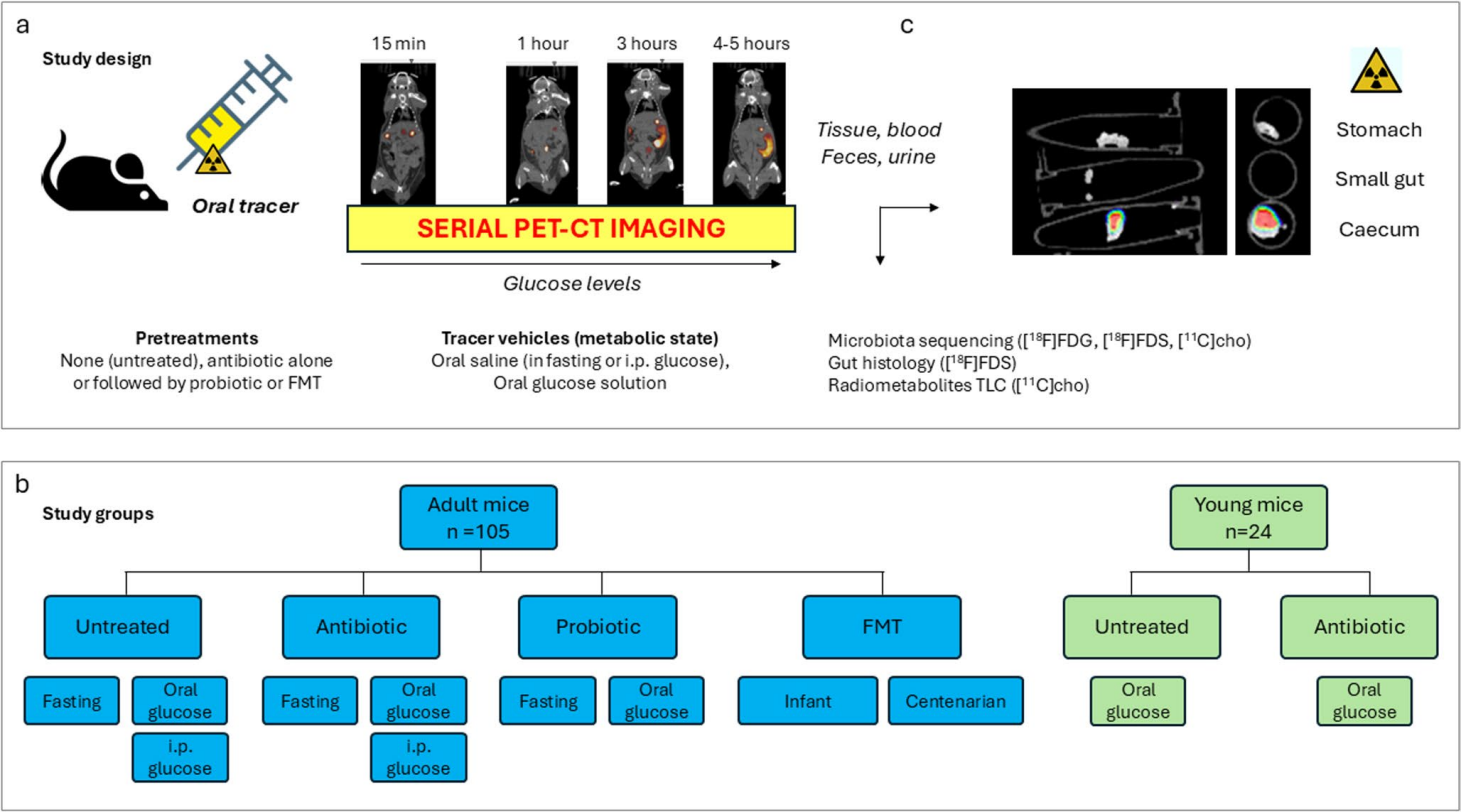
The study design (**a**) includes PET imaging of orally administered tracers in saline or glucose vehicles in untreated, antibiotic treated and probiotic or FMT treated mice, according to the study groups (**b**), whose subgrouping is further detailed in the text and in each figure legend. Repeated scans were acquired for 4–5 h, followed by ex vivo imaging of gut walls and contents (**c**), GM sequencing and tissue analyses, as appropriate

The study was conducted in adult and young post-weaning mice, housed in groups of min-max 2–4 mice per cage, under standard conditions of humidity of 50–60%, temperature of 22 °C, and daily cycles of 12 h of light and 12 h of darkness, with ad libitum access to food (standard diet in all groups) and water.

Sex-balanced groups were composed of 32 adult and 8 young mice (40 mice per each of three tracers: ∼120), each undergoing repeated whole-body PET-CT scans over four ([^11^C]) or five ([^18^F]) hours after oral tracer administration. In line with the available literature on oral [^18^F]FDS (*n* = 3/group) or oral ^64^Cu-*Bacteroides Fragilis* (*n* = 4/group) [19, 21], we estimated that subgroups of 4 mice were required to reach statistical power of 80% (alfa = 0.05), assuming an effect size, Cohen’s d = 3. Thus, our units of 40 mice were allocated as follows: (a) sixteen mice were left untreated (12 adults and 4 young mice); (b) sixteen were pretreated with antibiotics (12 adults and 4 young mice) to image the presence versus depletion of bacterial load along GI tracts. In each group of 12 adults, we used oral glucose vehicle (best reflecting dietary physiology, *n* = 4) or oral saline vehicle (with/without intraperitoneal glucose, *n* = 4 per condition) to exclude vehicle induced artefacts; (c) eight mice underwent oral probiotic inoculation (saline vs. glucose vehicle, *n* = 4 each) to examine differential tracer binding to a specific bacteria community. All subgroups were sex balanced.

Six additional adult mice receiving faecal microbiota transplantation (FMT) from two donors (infant vs. centenarian, 3 mice/donor, anonymized samples) were tested as surrogate hosts for human GM imaging, capturing physiological differences on an individual basis (personalized characterization).

After imaging, animals were euthanized, and samples of gut contents from seven GI tracts (to approach physiology of duodenum, jejunum, ileum, caecum, proximal, transversal, and distal sigmoid-rectal colon tracts) were collected for microbiome DNA extraction and sequencing by standard 16 S approaches. Selected tissues in animal subsets were used for histology of the small gut and caecum and circulating levels of hormones and inflammatory markers.

### Administration of antibiotics, FMT, and probiotics

In the antibiotic group, we administered 80 mg/kg clindamycin and 80 mg/kg enrofloxacin two times on the day before imaging (Day 1) and half dose few hours before imaging (Day 2). In probiotic and FMT groups, antibiotics pre-treatment (Day 1) was followed by two times implantation of probiotics or FMT (Day 2) and imaging (Day 3). The probiotic (commercial capsule-powder containing 50 billion live lactic acid bacteria per capsule, *Streptococcus thermophilus*, *Lactobacillus acidophilus*, *Bifidobacteria breve* and *animalis*) was dissolved in saline to obtain a concentrated solution of live bacteria (42 × 10^9^ in 0.6 ml) and a volume of 0.3 ml was administered via a sterile syringe, connected to a sterile cannula by oral gavage in the morning and evening of Day 2. For FMT, each frozen stool sample was thawed and diluted 1:10 w/v in sterile phosphate buffered saline (PBS) (0.1 g faecal sample in 1.0 ml PBS) and a volume of 0.2 ml of the suspension was administered (morning and evening of Day 2) by gavage.

### PET imaging procedure

Tracers in this study are commonly used in the clinical setting and were synthesized according to standardized and widely reported procedures [19, 22, 23]. After at least 4 h of fasting, mice were transported, through a clean cage, to the imaging laboratory. They were briefly sedated in an induction chamber with 3% isoflurane for measurement of basal glycemia from caudal blood using a commercial glucometer; then, the oral administration (plastic feeding tubes, 20 gauge x 30 mm, Instech FTP2030, 2Biological Instruments SNC, Besozzo VA, Italy) of tracer (~ 8 MBq ^18^F-tracers, ~ 14 MBq [^11^C]cho) ± glucose (2 mg/g of body weight by oral gavage or i.p. injection) in 250 µl of volume was carried out, keeping an upright position to avoid reflux. Mice were left awake and free to circulate in a cage with access to drinking water. During the imaging time, mice were sedated with isoflurane (1–2% v/v) and placed supine within the field of view of a small animal PET/CT scanner (IRIS PET/CT, Inviscan SAS, Strasbourg, France). PET images lasting 5–10 min were acquired at 15, 60, 180 and 300 min ([^18^F]FDG, [^18^F]FDS) or 15, 60, 120, 180 and 240 min ([^11^C]cho) after oral administration. Two added [^11^C]cho cases (for test and replication) underwent also dynamic scanning in the first 60 min, to confirm sufficiency of the above static scans to capture the rapid [^11^C]cho clearance. Short CT scans were performed to acquire anatomical images of each animal. Tail blood glucose levels were monitored by glucometer. At the end, mice were euthanized by anaesthetic overdose, and blood and tissues collected. PET data were corrected for dead time and radioactive decay and reconstructed by 3D-OSEM algorithm. Regions of interest were manually drawn on co-registered PET-CT images corresponding to stomach, proximal, central and distal small gut, caecum, proximal and distal colon, the bladder and relevant body organs, by using dedicated software (AMIDE Medical Image Data Examiner v.1.0.5). Activity levels were normalized by the injected dose per gram of body weight (%ID/g or standardized uptake value, SUV) reflecting the fractional tracer retention rate constant in bacteria and corrected for glucose levels in estimating the relative uptake of ingested glucose in body tissues.

### Radiometabolite analysis

Ingested choline undergoes catabolism with the formation of metabolites that contribute to its health-related effects, involving enterohepatic cycling and renal excretion. Considering the short half-life carbon-11, the distribution of [^11^C]cho and its radiolabelled metabolites were assessed exploratively in few separate mice, after 30–60 min of administration, in small gut, liver, gallbladder, urine. After methanol addition and tissue homogenization (~ 1.2 mL/g), samples were centrifuged (7 °C, 5 min, 14.000 rpm); an aliquot of the supernatant was applied to thin layer chromatography (TLC) plates (Silica gel 60 F₂₅₄, Merck), developed with acetonitrile/saline (60/40), and analysed by radio-TLC (miniGITA, Elysia Raytest) for metabolite identification [24].

### Ex-vivo microbiota characterization

Stool samples were collected in the same GI tracts analysed in PET images (stomach, proximal, central and distal small gut, caecum and proximal and distal colon). Microbial DNA was extracted using the QIAamp^®^ Fast DNA Stool Mini kit (QIAGEN, Hilden, Germany), as described [25]. The extracted DNA yield and quality were assessed with a NanoDrop ND-1000 spectrophotometer (NanoDrop Technologies, Wilmington, DE, USA). The V3-V4 hypervariable regions of the 16 S rRNA gene were amplified in 50 µl volume using KAPA HiFi HotStart ReadyMix PCR kit and the 341 F and 785R primers with Illumina adapter overhang sequences [26]. For library preparation, amplicons were purified using a magnetic bead-based system (Agencourt AMPure XP, Beckman Coulter, Brea, CA, USA) and indexed using Nextera technology through limited-cycle PCR. Indexed libraries were pooled at 4 nM, denatured with 0.2 N NaOH, and diluted to 5 pM. Sequencing was performed on an Illumina MiSeq platform using a 2 × 250 bp paired-end protocol according to the manufacturer’s instructions (Illumina, San Diego, CA, USA). Briefly, raw paired-end reads were quality-filtered and assembled using PANDAseq. Chimeric sequences were removed using USEARCH 11, and amplicon sequence variants, ASVs, were inferred using DADA2. Representative ASV sequences were then aligned and taxonomically assigned within QIIME 2 version 2023.5 using VSEARCH-based/hybrid alignment against the SILVA database (SSU Ref NR v.138). The ASV table was rarefied prior to downstream diversity analyses. Taxonomic profiles were then used to generate relative-abundance tables, while alpha- and beta-diversity metrics were computed in QIIME 2. Beta diversity was calculated with the “qiime diversity core-metrics-phylogenetic” plugin and estimated by computing weighted and unweighted UniFrac distances, which were used as input for principal coordinates analysis, PCoA. ASVs were filtered for a prevalence of ≥ 0.2% in at least 1/6 of all samples and used to generate plots with the R packages “vegan” (http://www.cran.r-project.org/package=vegan) and “MicrobAIDer” (https://github.com/FabbriniMarco/microbAIDeR).

### In vitro tracer uptake by bacteria

To corroborate imaging versus bacteria correlations, we performed in vitro testing by exposing cultured bacteria to our probes. Since most gut commensals cannot be grown, we cultured bacteria from commercially available probiotics belonging to the taxonomic families emerging in this study, namely *Clostridiaceae* (*Clostridium butyricum* for [^18^F]FDG) and *Lactobacillaceae* (*Pediococcus acidilactici* and *Lactobacillus plantarum* for [^11^C]cho). More details are provided in the legend of Supplementary Information S4.

### Gut histology

Histology was performed in small gut and caecum samples obtained from 29 animals to evaluate if [^18^F]FDS urinary excretion reflects gut damage, focusing on the mucosa, submucosa, and muscularis externa layers. In the mucosa, crypts (and villi in the small intestine) and goblet cells were counted in each image. Three crypts or villi per image were analysed at 20× magnification for apical and basal length, total length, width and transversal area. Additionally, the depth and number of open spaces between crypts were measured. The area of three goblet cells per image was quantified at 40 × magnification. The thickness of the submucosa and muscularis externa (including circular and longitudinal sublayers) was assessed in three zones per image and averaged.

### Serum markers

Available samples of adult mice (*n* = 74) were assayed for circulating levels of inflammatory markers IL-6, TNF-α, GI peptides, adipokines and metabolic hormones (insulin, C-peptide), using a multi-analyte panel based on Luminex^®^ xMAP^®^ technology (Milliplex map kit, CAT N# MMHMAG-44 K, Merk Life Science S.r.l. Milan, Italy).

### Statistical analyses

Data are shown as mean ± sem, and differences were detected by analysis of variance (metabolic variables) or Kruskal-Wallis and Wilcoxon tests (GM), unless otherwise specified. Spearman’s regression analyses were performed to correlate metabolic and GM parameters. All GM related correlations and comparisons were also examined after false discovery rate (FDR) correction and both uncorrected and corrected results are shown. Likewise, main PET kinetics data achieving inter-group differences in single point comparisons (Figures) were also tested with more restrictive post-hoc evaluations (Supplementary Table 1). A value of *p* < 0.05 was considered as statistically significant.

## Results

We analysed ~ 170 [^18^F]FDG and ~ 170 [^18^F]FDS images (43 cases per tracer, 4 PET scans each), and 220 [^11^C]cho images (44 cases, 5 PET scans each), each including stomach, proximal, central and distal small gut, caecum, proximal and distal colon, bladder and other relevant organs, resulting in > 7000 regions of interest drawn by a blinded operator.

### Functional GM PET imaging in adult mice

**Fig. 2:**
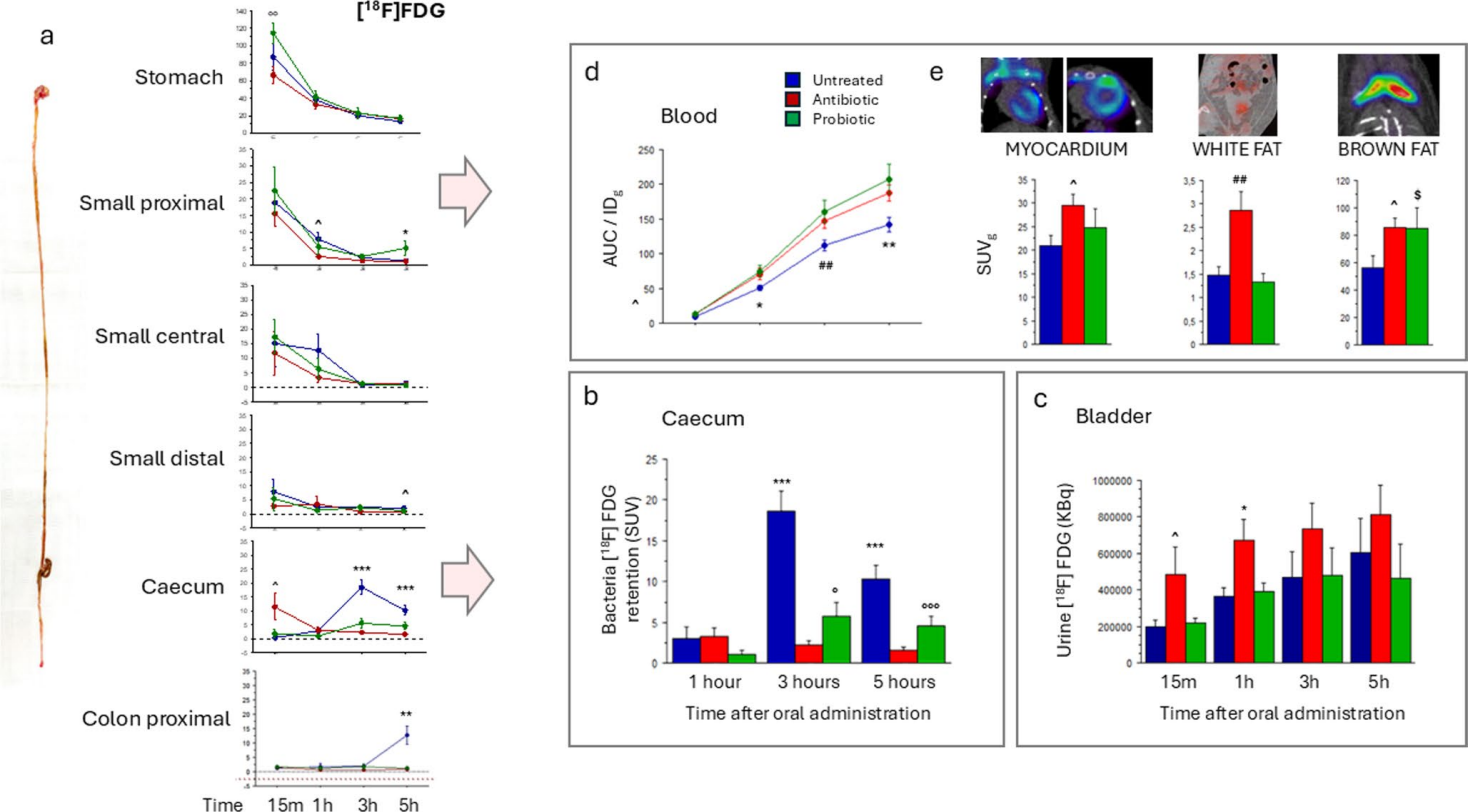
Time-course of [^18^F]FDG in different segments of the GI tract (**a**), highlighting significant accumulation in the caecum and proximal colon in untreated mice (blue line), as fully suppressed by antibiotics (red line) and partially restored by probiotics (green line), indicative of bacteria retention. Uptake in the proximal small intestine suggests probiotics colonization of this niche. The other panels show: (**b**) magnified group comparisons of [^18^F]FDG in caecum, (**c**) urine [^18^F]FDG excretion, (**d**) appearance of oral [^18^F]FDG in blood and (**e**) the uptake of dietary glucose in relevant body organs, as increased by antibiotics. Groups are composed of 12 untreated, 12 antibiotic and 8 probiotic treated mice; data are given as mean and standard error; analysis of variance (or unpaired t-test for ° and °°°) ***p ≤ 0.0001, **p ≤ 0.001, *p < 0.05, ^##^*p* ≤ 0.01 vs. all groups; ^*p* < 0.05 untreated vs. antibiotic; °°°*p* = 0.02, °°*p* = 0.03, °*p* = 0.057 probiotic vs. antibiotic; ^$^*p* < 0.057 probiotic vs. untreated

**Fig. 3:**
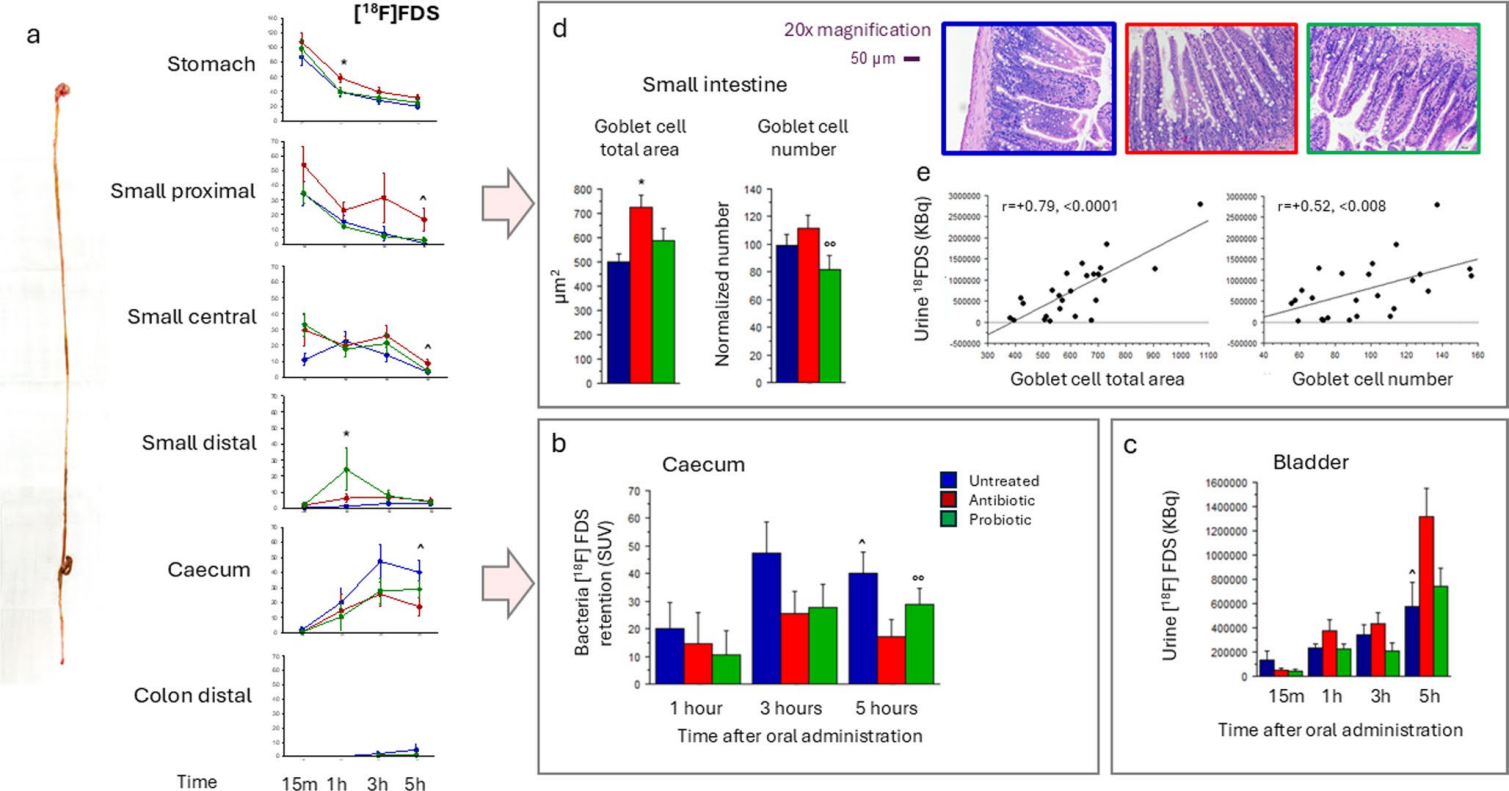
Time-course of [^18^F]FDS in different segments of the GI tract (**a**), highlighting significant accumulation in the caecum in untreated mice (blue line), as partly suppressed by antibiotics (red line) and partially restored by probiotics (green line), consistent with bacterial uptake and intracellular trapping of the tracer. Notably, antibiotic treatment results in the enrichment of [^18^F]FDS avid bacteria in the small intestine. The other panels show: (**b**) magnified group comparisons of [^18^F]FDS in caecum, (**c**) urine excretion, as increased in antibiotic groups, and (**d**) alterations in small gut walls histology in antibiotic groups, (**e**) correlating with the accumulated urinary [^18^F]FDS. Groups are composed of 12 untreated, 12 antibiotic and 8 probiotic treated mice (PET), and 7–9 mice per group (histology); data are given as mean and standard error; analysis of variance *p < 0.05 vs. all groups; ^*p* < 0.02 untreated vs. antibiotic; °°*p* < 0.05 probiotic vs. antibiotic

**Fig. 4:**
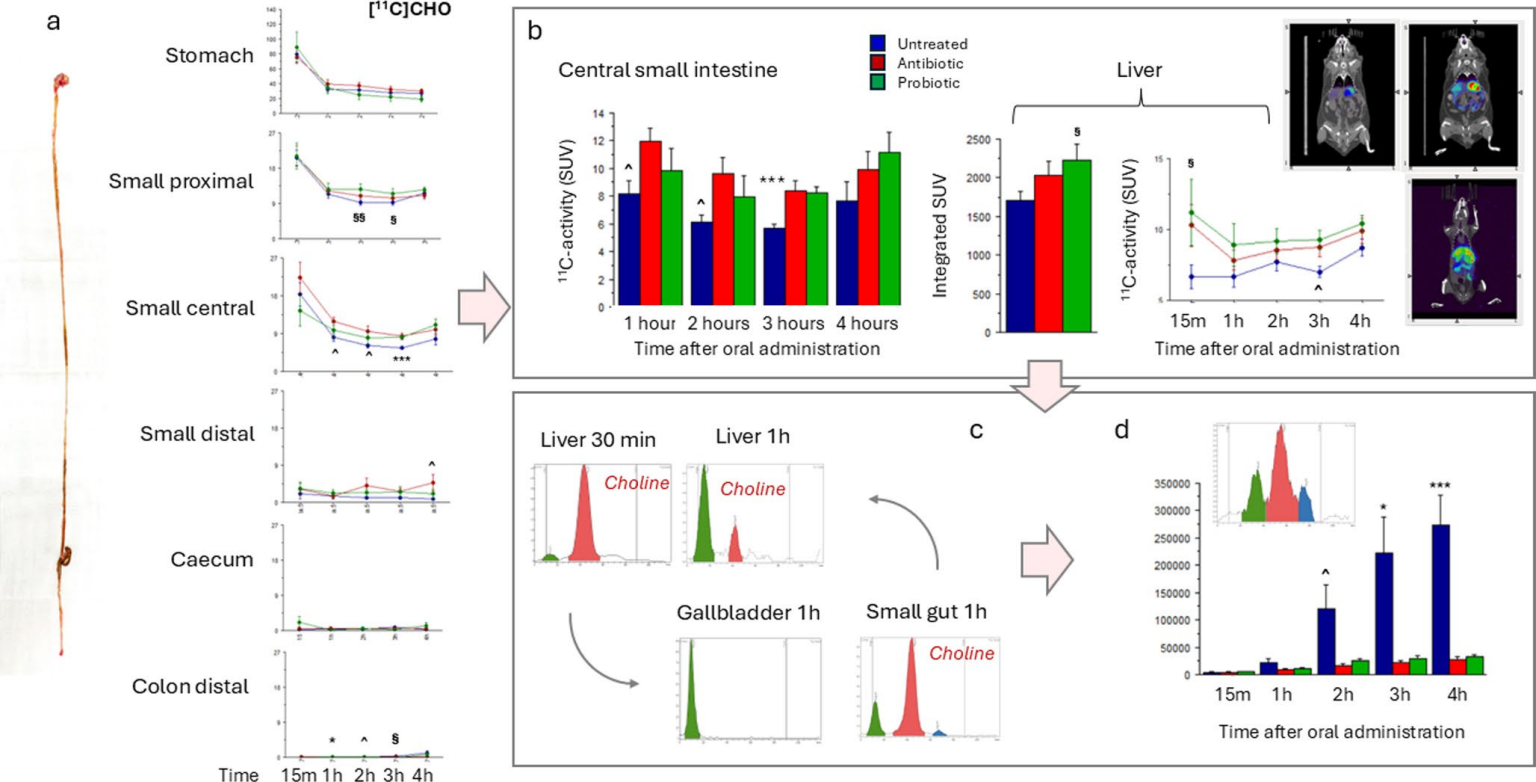
Time-course of [^11^C]cho in different segments of the GI tract (**a**), highlighting the rapid absorption of ^11^C from proximal-central small gut into the liver and body in untreated mice (blue line); such clearance was significantly blunted by antibiotics (red line) resulting in higher ^11^C levels in the distal small intestine, and minimally restored by probiotics (green line). The other panels show: (**b**) magnified group comparisons of [^11^C]cho in central small gut and liver, with images clearly showing the enterohepatic cycle, as confirmed by (**c**) the radiometabolites appearing in liver, gallbladder and small gut, and (**d**) the urinary excretion of activity only in untreated mice, as nearly abolished by antibiotics. The marked suppression of intestinal clearance and urinary excretion of ^11^C-activity by antibiotic treatment supports a GM-dependent contribution to early choline handling, despite the known involvement of hepatic metabolic pathways. Groups are composed of 14 untreated, 12 antibiotic and 8 probiotic treated mice; data are given as mean and standard error; analysis of variance ***p ≤ 0.0005, *p < 0.05 vs. all groups; ^§§^*p* < 0.01, ^§^*p* < 0.05 untreated vs. probiotic; ^*p* < 0.05 untreated vs. antibiotic

The kinetics of each tracer in adult mice is shown in respective Figs. 2, 3 and 4, with multivariate corrections given in Supplementary Information Table 1. The distribution of [^18^F]FDG along GI tracts (Fig. 2a-b) documented a rapid progression in-and-out of the stomach and the small intestine, with approximately 70% of the initial activity being washed-out at 1 h and > 90–95% at 3–5 h after ingestion. Conversely, [^18^F]FDG underwent remarkable trapping at the level of caecum contents in untreated animals, accumulating by 20–40 folds at later (vs. early) imaging time points. There was some smaller but still progressive accumulation also in the proximal colon. The opposite kinetics was observed in antibiotic treated animals, in which [^18^F]FDG was washed out of caecum and colon, with no accumulation (~ 12–15% compared to the signal seen in untreated mice, *p* < 0.0001). The absence of selective [^18^F]FDG retention due to antibiotics indicates that glucose fermenting bacteria are mostly operative in the caecum, followed by distal small and proximal large gut in the 5-hour timeframe of the study. In mice receiving probiotics to repopulate the gut after antibiotic-depletion, [^18^F]FDG kinetics showed a significant degree of accumulation over time, and the [^18^F]FDG signal exceeded by 3 times the uptake of [^18^F]FDG occurring in antibiotic treated mice (*p* < 0.05), remaining considerably lower than seen in untreated mice (*p* = 0.003). In the small intestine, instead, the probiotic signal was significantly greater than in untreated mice. These different patterns support our hypothesis that PET imaging can chase bacteria and their sites of dominant engraftment and action based on tracer kinetics.

The distribution of [^18^F]FDS (Fig. 3a-b) displayed some resemblance, but also notable differences compared to [^18^F]FDG. Like [^18^F]FDG, it presented progressive (even higher) accumulation in caecum contents, with twice as high retention values. However, the suppression of [^18^F]FDS retention in antibiotic treated compared to untreated mice in the caecum was less pronounced than seen for [^18^F]FDG, and it was accompanied by significant [^18^F]FDS uptake in the small intestine. These observations suggest that [^18^F]FDS-consuming bacteria may be resilient and even able to expand in selected gut niches under conditions of microbiota depletion. The oral glucose vs. saline vehicle reduced the [^18^F]FDS, but not the [^18^F]FDG PET signal in the caecum (Supplementary Information S1), suggesting that glucose and its analogue are preferred substrates of bacteria in this region.

The kinetics of [^11^C]cho is shown in Fig. 4a-b, denoting that the metabolism and body absorption of choline occurred primarily in the small gut, with negligible amounts of radioactivity reaching subsequent intestinal tracts. Images of [^11^C]cho document a visible enterohepatic cycle, consistent with the rapid absorption of [^11^C]activity from the proximal and central small gut into the liver, with plateauing gut-liver activity over time. Here, the antibiotic group showed significantly slower clearance of [^11^C]activity from small gut and liver, with higher activity transiting into the distal small intestine.

### Functional PET imaging differences between maturing and mature microbiota

**Fig. 5:**
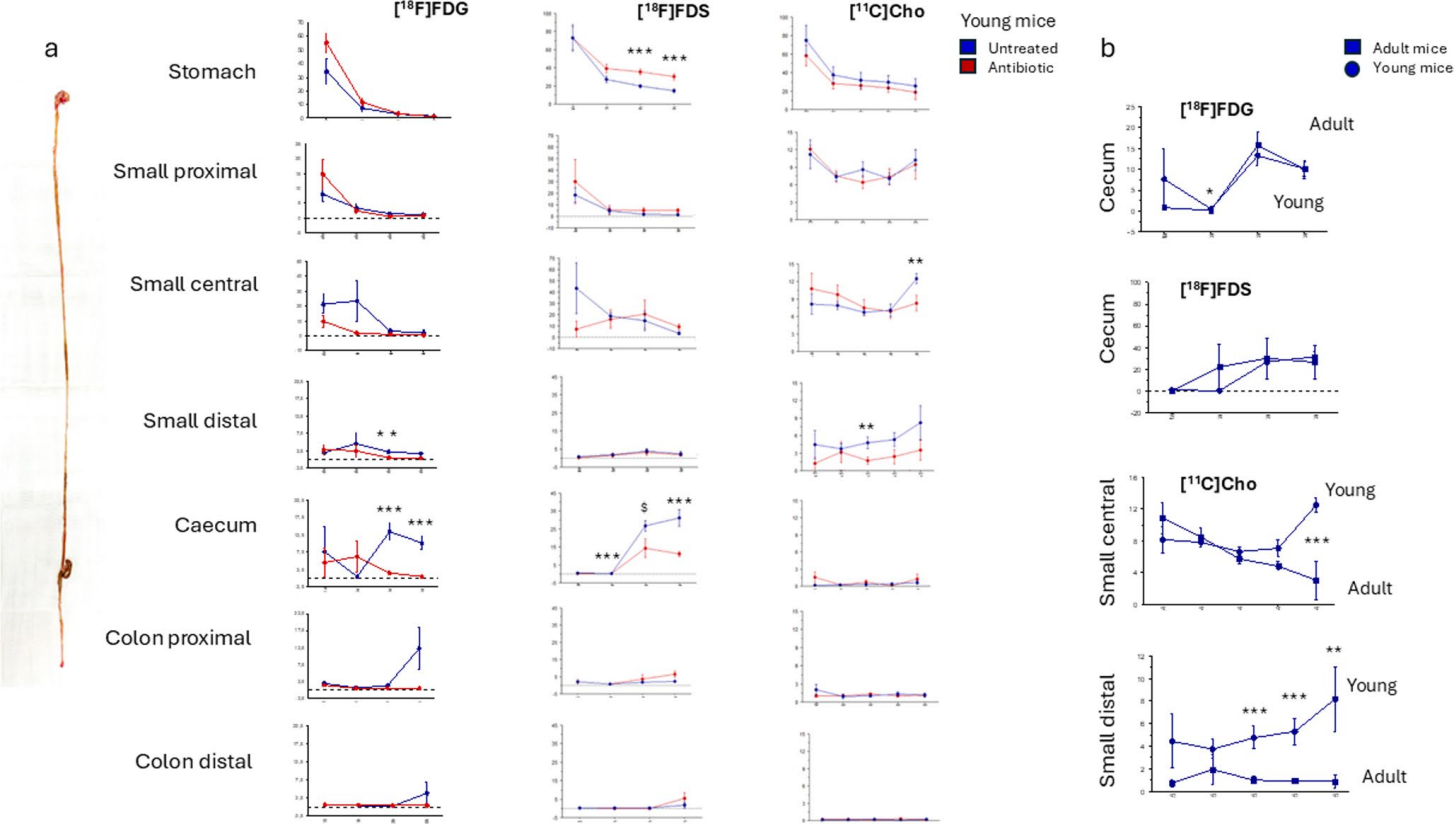
In young post-weaning mice, the time-course (**a**) of [^18^F]FDG and [^18^F]FDS showed accumulation in the caecum and significant suppression by antibiotics, whereas [^11^C]cho metabolism by the immature GM was unaffected by antibiotic. Compared to untreated adults (**b**), in young mice the kinetics of [^18^F]FDG and [^18^F]FDS was similar, whereas much greater proportions of [^11^C]cho remained in the central gut and transited to the distal gut. Groups of young mice are composed of 8 untreated and 8 antibiotic treated mice; data are given as mean and standard error; analysis of variance ***p ≤ 0.001, **p ≤ 0.04, *p < 0.05, ^$^*p* = 0.08 between groups

The kinetics of [^18^F]FDG and [^18^F]FDS was similar, whereas [^11^C]cho dynamics was radically different in young compared to adult mice (Fig. 5). Young untreated mice showed significant accumulation of [^18^F]FDG and [^18^F]FDS in the caecum, and marginally in the colon over time, with strong antibiotic-induced suppression of the PET signal, thus reproducing the patterns seen in adult mice (Fig. 5a). Compared to adults, absolute retention values of [^18^F]FDG and [^18^F]FDS in young mice were not significantly lower in the caecum (Fig. 5b). Results of [^11^C]cho kinetics suggest an immature contribution of GM to choline metabolism in young mice, lacking the response to antibiotic seen in adult mice (Fig. 5a). Consistently, young versus adult mice showed progressive build-up of ^11^C-activity in the central and distal small intestine over time, denoting inefficient metabolism and body clearance (Fig. 5b).

As shown in Supplementary Information Table 1, multivariate statistics was mostly confirmatory of the results given in Figs. 2, 3, 4 and 5; in the sole [^18^F]FDS adult group, differences in the overall kinetics were weakened, due to higher intersubject variability, as visible in Fig. 3a-b. 

### Functional PET imaging is coherent with the abundance of functionally correlated bacteria

**Fig. 6:**
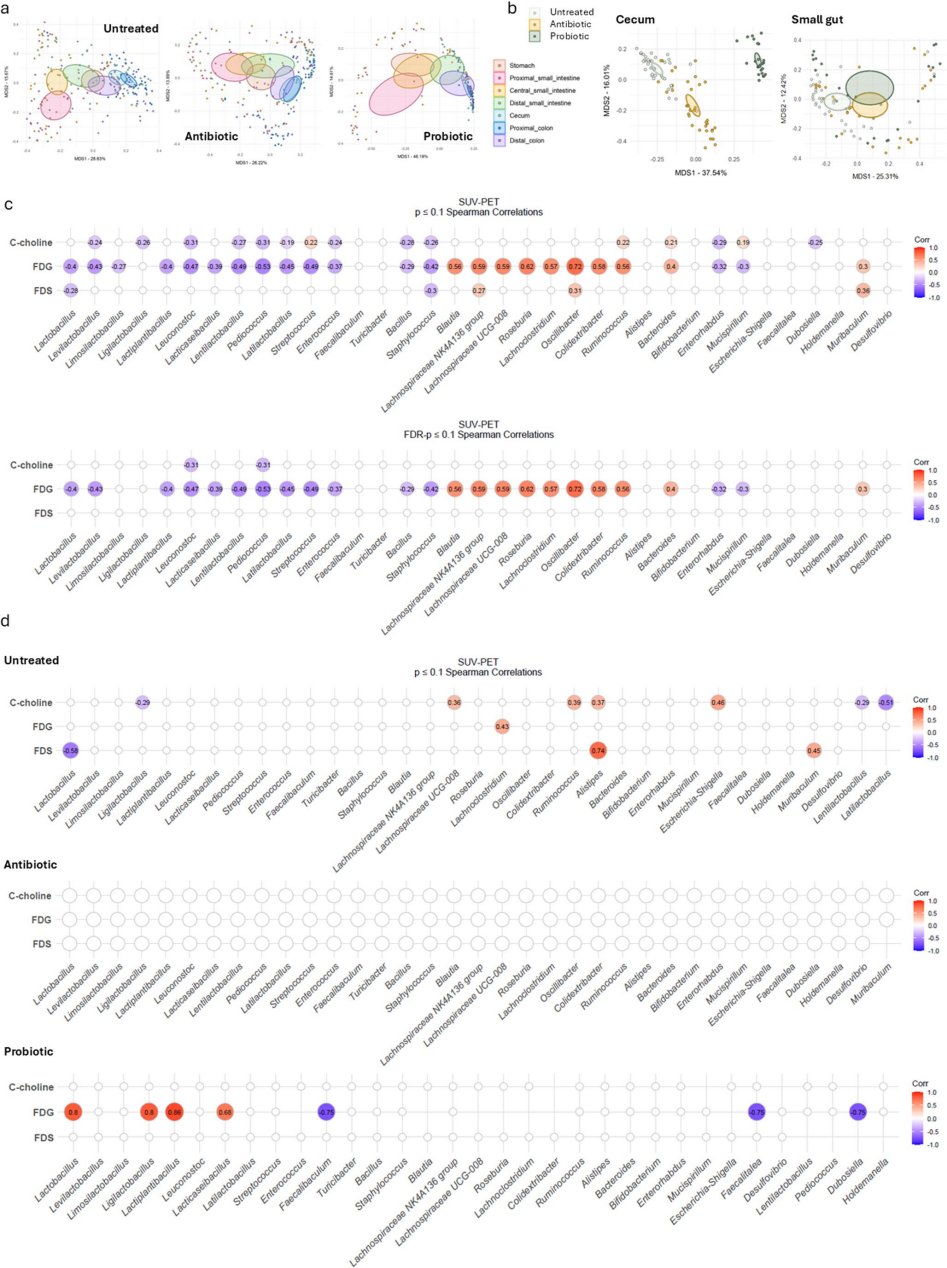
Segment-specific gut microbiota composition and its association with functional PET imaging signals. β-diversity analysis (Bray-Curtis) (**a**) demonstrates marked compositional dissimilarity (*p* < 0.0001) between GI segments across all experimental groups, highlighting the strong spatial structuring of the gut microbiota. Significant treatment-dependent differences in β-diversity (**b**) are observed in intestinal segments emerging as dominant sites of tracer handling, namely caecum for [^18^F]FDG and [^18^F]FDS, and proximal-central small intestine for [^11^C]cho. Heatmap and regression coefficients (**c**) illustrate significant associations between PET tracer retention ([^18^F]FDG and [^18^F]FDS in caecum; [^11^C]cho in proximal-central small intestine) and the relative abundance of bacterial genera in pooled treatments. Associations reflect community-level functional enrichment rather than species-specific metabolic attribution. Stratified analyses (**d**) by treatment group (untreated, antibiotic-, probiotic-treated) show loss of significant associations between PET data vs. relative abundance of bacterial genera following microbiota depletion by antibiotics and context-dependent re-emergence of tracer-taxa relationships after probiotic replacement

We collected 910 samples for GM characterization in the seven intestinal segments analysed in PET images in all mice (stomach, proximal, central and distal small gut, caecum, proximal and distal colon). Optimal quality of the extracted material was obtained in 773 (85%) samples. Results confirmed that GM composition (β-diversity, Fig. 6a) is very distant between gut tracts, with greater similarities within (than between) the small and large intestines. Highly significant distances between untreated, antibiotic and probiotic treated groups were observed in the intestinal tracts emerging as main bacterial functional sites from our PET images, namely the caecum for [^18^F]FDG and [^18^F]FDS and the proximal and central small gut for [^11^C]cho (Fig. 6b, Supplementary Information S1a, S1c, S1g). Instead, β-diversity (Bray-Curtis plots) and microbiota taxa (stacked bar chart) were similar between oral vehicles (saline, glucose solution, Supplementary S1b, S1d-g), allowing their pooling in regression analyses.

We examined whether the PET signal of each tracer reflects the predominance of given bacteria in the emerging target segments (caecum and small intestine) (Fig. 6c), before and after correction for False Discovery Rates (FDR). Supplementary Information S2-S3 show corresponding group differences in the abundance of all correlating bacteria, without (S2) and with FDR adjustment (S3). We were interested in bacteria, whose enrichment would be associated with higher [^18^F]FDG and [^18^F]FDS levels in the caecum (positive relation) and with lower levels (= greater metabolism) of [^11^C]cho in the proximal-central small intestine (inverse relation). Given the taxonomic resolution of 16 S rRNA sequencing, these associations should be interpreted as a community-level functional enrichment rather than strain-specific metabolic attribution. Results clearly indicated that [^18^F]FDG retention in the caecum was strongly and selectively associated with the relative abundance of multiple bacterial genera, predominantly belonging to the *Clostridia* class and, to a lesser extent, the *Bacteroidia* class. These associations remained robust after FDR correction, suggesting that these communities represent the dominant glucose-utilizing bacterial fraction in the murine caecum. [^18^F]FDS uptake in the caecum was related with a smaller share of (two) *Clostridia* class bacteria genera, and with the *Muribaculum* genus. Finally, lower levels of [^11^C]cho in the proximal-central small intestine were predictive of higher abundances of 11 bacteria genera, 8 belonging to the Bacillota phylum, including 7 in the *Bacilli* class (6 in the *Lactobacillales* order, 2 in the *Bacillales* order) and *Dubosiella (Erysipelotrichales* order*)*, the remaining being *Enterorhabdus* (Actinomycetota phylum). Among them, *Pediococccus* and *Leuconostac* genera (*Lactobacillales* order) survived FDR correction. Supplementary Information S4 shows high and persistent retention of [^18^F]FDG in cultured *Clostridium butyricum* and [^11^C]cho in cultured *Lactobacillales* with negligible uptake seen in heat-killed bacteria vs. control broth.

Notably, [^18^F]FDG retention in the caecum was inversely associated with the relative abundance of several *Lactobacillales* genera, as well as *Bacillus*,* Enterococcus*,* Staphylococcus*, and *Enterorhabdus*. This pattern suggests a functional hierarchy within the intact microbiota, whereby saccharolytic, SCFA-producing communities, particularly *Clostridia*, may outcompete lactic acid-producing bacteria for glucose-derived substrates. Figure 6d shows separate correlations in each treatment group, highlighting that following antibiotic-mediated depletion and selective probiotic replacement, [^18^F]FDG uptake became strongly and positively associated with *Lactobacilli* abundance, reflecting a context-dependent reorganization of microbial metabolic dominance. Figure 6d also shows the lack of correlation of any tracer in the antibiotic group, as well as the absence of correlations of [^18^F]FDS and [^11^C]cho with probiotic group bacteria (*Bifidobacteria*, S*treptococcus thermophilus* and *Lactobacilli*), reinforcing PET imaging findings of a neutral effect of the probiotic on the kinetics of these tracers.

Finally, we examined the correlation of PET values for each tracer in all GI tracts and corresponding GM abundances in all GI tracts (Supplementary Information S5) and found the known affinity of *Enterobacteria* (*Escherichia*-*Shigella*) for [^18^F]FDS.

### Functional PET imaging can predict FMT composition

**Fig. 7:**
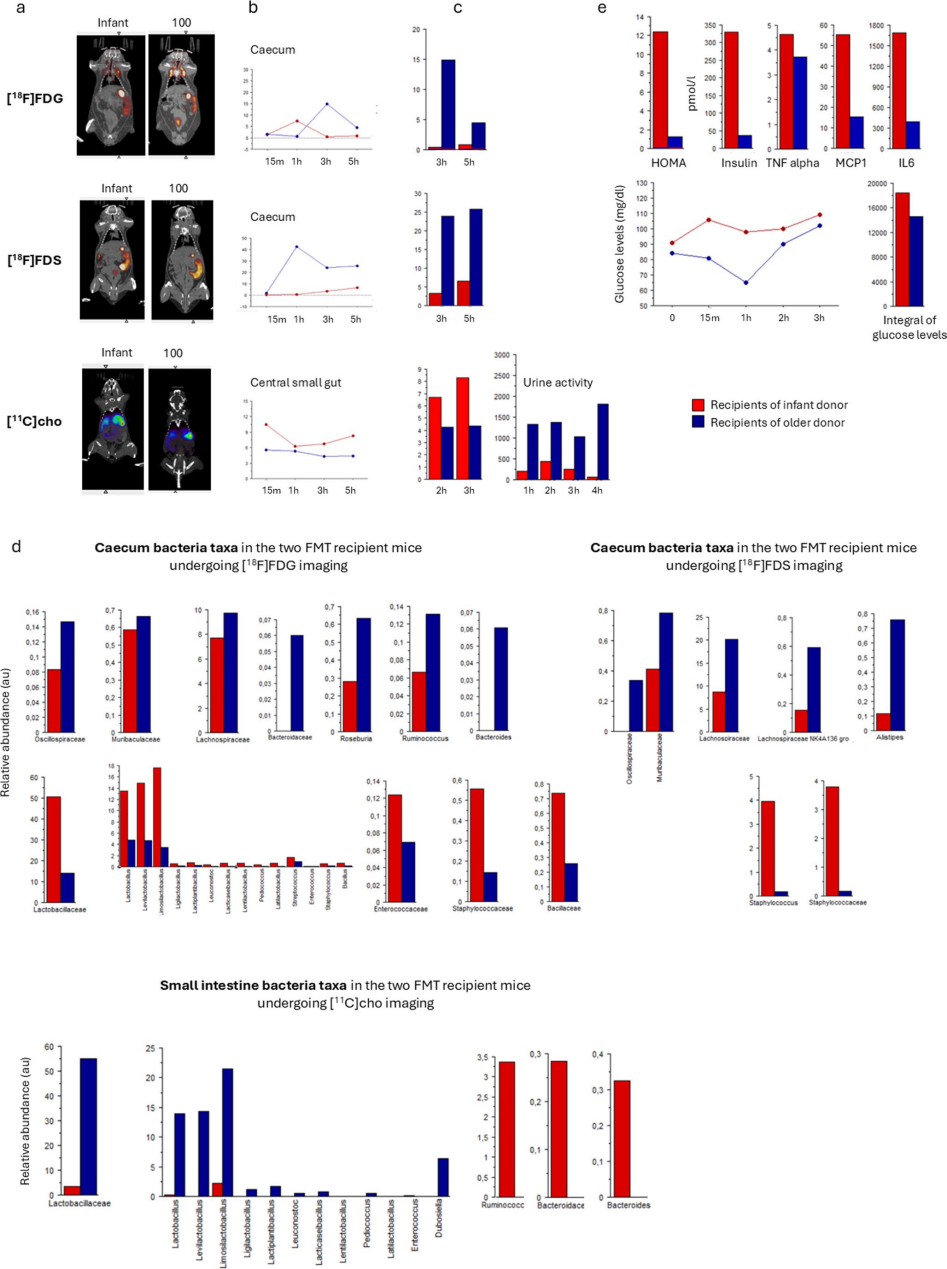
PET imaging discriminates donor-associated microbiota functional phenotypes following faecal microbiota transplantation (FMT). Representative PET images (**a**) of mice receiving FMT from an infant or a centenarian human donor. Time-activity curves of [^18^F]FDG, [^18^F]FDS, and [^11^C]cho (**b**-**c**) in recipient mice show distinct donor-associated tracer kinetics, with greater caecal retention of [F]-labelled tracers and faster intestinal clearance of [^11^C]cho in recipients of the centenarian GM compared with recipients of the infant GM. Ex vivo microbiota sequencing (**d**) in caecum and small intestine reveals donor-associated differences in bacterial community composition that are consistent with the observed functional imaging patterns. High abundance of *Clostridia* and *Bacteroidia* in caecum and *Lactobacillaceae* in small gut in old donor’s recipients justifies the high [^18^F]FDG and [^18^F]FDS retention and rapid [^11^C]cho clearance in respective recipients, while high *Lactobacillaceae* and *Staphylococcaceae* in caecum and *Ruminococcaceae*, *Bacteroides*, *Bacteroidaceae* in small gut are in agreement with the opposite tracers kinetics emerging from the younger donor’s FMTs. The metabolic profile was assessed in two representative FMT recipients (**e**) after [^11^C]cho imaging supporting the physiological relevance of the imaging-derived GM-associated phenotypes

To test whether functional PET imaging can resolve individualized microbiota-associated phenotypes, we performed FMT from two human donors at opposite ends of the age spectrum (infant and centenarian) into antibiotic-pretreated adult mice. Each donor microbiota was transplanted into three recipient mice, which subsequently underwent PET imaging, each with one tracer ([^18^F]FDS, or [^18^F]FDG, or [^11^C]cho) (Fig. 7a-c). FMT resulted in markedly distinct tracer kinetics between recipient groups. Mice receiving microbiota from the centenarian donor showed progressive and sustained accumulation of both [^18^F]FDS and [^18^F]FDG in the caecum, as opposed to minimal retention seen in the immature FMT of the infant donor. Likewise, the clearance of [^11^C]cho by the mature than immature microbiota was twice as high, and urine radioactivity many folds higher. These patterns indicate that imaging FMT maps are highly individualized. We tested if the [^18^F]FDS, [^18^F]FDG and [^11^C]cho PET imaging data seen in FMT mice were consistent with the correlations observed in Fig. 6. In fact, ex vivo microbiota sequencing in Fig. 7d revealed compositional differences consistent with the observed imaging phenotypes, as caecum *Clostridia* and *Bacteroidia* genera were enriched in mice receiving FMT from the old donor, coherent with high uptake of [^18^F]FDS, [^18^F]FDG, whereas *Lactobacilli* prevailed in caecum of mice receiving FMT from the infant donor, in line with the low uptake of [^18^F]FDS, [^18^F]FDG. Moreover, [^11^C]cho results agreed with Fig. 6 in the abundance patterns of *Lactobacilli* and *Enterococcus*, *Staphylococcus*, *Enterorhabdus* and *Dubosiella* genera in the central small intestine of recipients.

### PET imaging of gut-body crosstalk

Untreated mice transferred a lower proportion of “dietary” [^18^F]FDG in the circulation, as compared to microbiota-depleted mice (Fig. 2d), suggesting that bacteria sequester glucose for their metabolism, preventing its absorption. The elimination of [^18^F]FDG in urine was not significantly different between groups at later time-points (Fig. 2c). Instead, we observed that the fraction of dietary glucose (SUV_g_) being extracted by the myocardium, brown and white fat was higher in treated groups (Fig. 2e), suggesting that the lack of microbiota-derived energy substrates may preferentially direct glucose towards organs that are continuously working (heart) or dependent on glucose (fat), and the probiotic seemed to normalize the uptake only in white fat. This is consistent with prior evidence in germ-free mice undergoing isolated heart perfusion experiments [27].

Images confirmed that [^18^F]FDS was (expectedly) not extracted by body organs, and the absorbed proportion was eliminated in urine, supporting the potential of urine-[^18^F]FDS-imaging (bladder) to probe intestinal integrity. In the available samples, we examined the caecum and small intestine and found that the total area and number of goblet cells observed in the small gut were enlarged in antibiotic treated mice (Fig. 3d), correlating with the amount of [^18^F]FDS found in urine (Fig. 3e). In the caecum, the muscularis wall was thinned in antibiotic treated mice (Supplementary Information S6a-b), and goblet cells and crypts were expanded in probiotic treated mice (S6c-d).

In the [^11^C]cho study, we found ^11^C-activity in urine (bladder) images in untreated mice, which was nearly absent in bacteria-depleted groups, as induced by antibiotics, whether followed or not by probiotics (Fig. 4d). These findings denote an important effect of bacteria on choline metabolism and fate. Our explorative characterization of radiometabolites (Fig. 4c-d**)** showed that [^11^C]cho was progressively reduced in favour of one dominant ^11^C-metabolite in the liver, flowing in the small gut via the gallbladder, undergoing recirculation and urinary excretion. We tested two antibiotic treated mice (Supplementary Information S7), observing the same ^11^C-species, but with low hepatic formation and absent urine excretion of the major metabolite. One smaller ^11^C-metabolite peak appeared in the small gut content in one of two untreated cases and in liver and gallbladder in one of two antibiotic treated cases. Based on the literature, these findings suggest the major peak to be betaine, and the intestinal peak might be trimethylamine, consistent with the observation of urinary methylamines reported in rats ingesting ^14^C-cho as nutritional supplement [28, 29].

### Functional PET imaging of microbiota as biomarker of insulin-resistance and inflammation

**Fig. 8:**
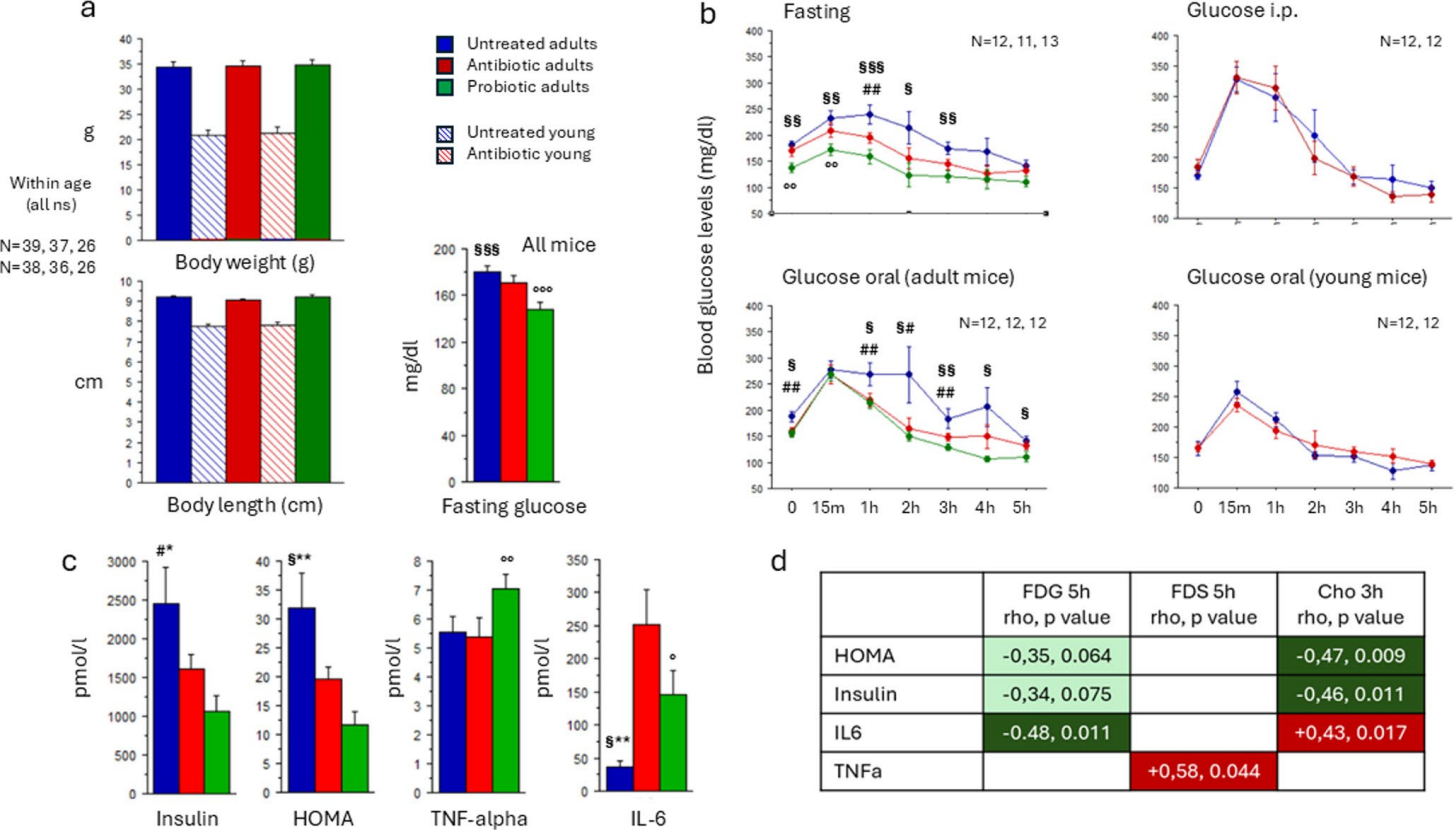
Body weight and length (**a**) were matched within young and adult groups. Probiotics and to a lesser extent antibiotics showed lower glucose levels (**b**) especially in adult fasted mice and mice receiving oral glucose and higher insulin sensitivity (**c**) compared to untreated mice. Untreated mice showed the lowest degree of inflammatory indicators (**c**), with antibiotic treatment presenting highest levels, and probiotic replacement being intermediate. PET imaging of [^18^F]FDG, [^18^F]FDS and [^11^C]cho predicted inflammation and insulin sensitivity (**d**), with high [^18^F]FDG uptake being protective from insulin resistance (HOMA, insulin) and inflammation (IL-6), high [^18^F]FDS uptake being proinflammatory (TNF-α), and high [^11^C]cho levels (poor gut and urine clearance) being proinflammatory (IL-6), but protective from insulin resistance. Groups in Panels c-d refer to 26 untreated, 27 antibiotic and 21 probiotic treated mice; numbers in the other groups are given in respective graphs; data are given as mean and standard error, and compared by analysis of variance **p ≤ 0.001, *p < 0.005, ^##^*p* ≤ 0.05, ^#^*p* = 0.06 untreated vs. antibiotic; ^§§§^*p* < 0.0002, ^§§^*p* < 0.005, ^§^*p* ≤ 0.05 untreated vs. probiotic; °°°*p* < 0.005, °°*p* < 0.05, °*p* = 0.06 probiotic vs. antibiotic; Spearman’s regression coefficients are given in d

The metabolic profile of study groups is shown in Fig. 8. Age-matched mice had similar body weight between treatments (Fig. 8a). Glucose levels were lower in the antibiotic or probiotic treated than untreated groups, under fasting and oral glucose loading, consistent with the higher organ partition of dietary glucose observed in [^18^F]FDG images (Fig. 8b). In regression analyses, [^18^F]FDG uptake in caecum bacteria was reflective of better health, namely lower levels of insulin resistance and proinflammatory IL-6, whereas [^18^F]FDS was predictive of greater levels of proinflammatory TNF-α. Low [^11^C]cho values in the small gut as seen in untreated mice, were associated with high insulin resistance and low levels of IL-6 (Fig. 8d). The resulting balance led to lower inflammatory and higher metabolic risk in untreated than antibiotic treated mice, with best metabolic profile in probiotic replaced groups (Fig. 8c). The metabolic profile was also assessed in one representative FMT recipient per infant and per older donor (Fig. 7e), showing that imaging signatures of immature GM translate in high levels of glucose, insulin, insulin resistance, and proinflammatory markers.

## Discussion

What enters our digestive tract will unavoidably be in contact with gut bacteria, affecting the resulting crosstalk with body metabolism. The current results open a new perspective for PET imaging to become a mainstay of patients’ GM-based stratification and counselling, considering the relevant role played by GM in a multitude of common diseases, including obesity, diabetes, cardiovascular and immune diseases, neurological and psychiatric disturbances, cancer and the response to cancer therapy. Unlike metagenomic or metabolomic approaches, which provide static or ex vivo snapshots of microbial potential or metabolic output, PET imaging enables a dynamic, spatially resolved assessment of microbial activity within its native intestinal niche. This approach captures the integrated outcome of microbial abundance, substrate accessibility, competitive interactions, and host modulation, thereby complementing sequencing-based methods and offering unique translational potential. Our data demonstrate that the imaging of orally administered probes is simple and effective to map GM characteristics in situ and the functional crosstalk with body processes in mice. Our data confirm that the GI ecosystem is highly diversified, pointing to the small intestine and caecum as predominant players in gut-body handling of our target nutrients.

The current study documents that [^18^F]FDG and [^18^F]FDS (unmetabolizable glucose and sorbitol analogues) undergo lasting retention in the caecum, as major site of fermentation in mice; [^11^C]cho undergoes metabolism by bacteria in the small gut, followed by enterohepatic circulation and urinary excretion.

The observed caecum accumulation of [^18^F]-sugar analogues over time indicates that they were trapped in bacteria, consistent with the notion that these tracers cannot be metabolized and remain trapped intracellularly in their phosphorylated form, as opposed to [^11^C]cho, undergoing catabolism and recirculation. Large spectrum antibiotic pre-treatment was used to deplete GM and capture where and to what extent the PET signal was due to bacteria. In fact, this intervention suppressed [^18^F]FDG and [^18^F]FDS uptake primarily in the caecum and [^11^C]cho clearance in the small intestine. The administration of a probiotic after antibiotic pretreatment (replacing selected bacteria) showed partial reversion of antibiotic induced results, consistent with its specific bacteria composition. Our findings agree with a previous report showing similar reactions of caecum [^18^F]FDS to antibiotic and probiotic.

To our knowledge, this study provides first evidence of a relationship between GI mapping of oral PET probes and corresponding measures of bacteria abundances, as obtained by gold-standard ex-vivo rDNA sequencing. The imaging signal of [^18^F]FDG, [^18^F]FDS and [^11^C]cho was proportional to tracer-specific communities of fermenting or choline-metabolizing bacteria abundances.

The most robust correlations were found between [^18^F]FDG binding to caecum bacteria and the abundance of bacteria genera belonging to *Clostridia* and *Bacteroidia* classes, which is entirely consistent with the known physiology of these commensal bacteria as main sources of short-chain fatty acids (SCFAs) from sugar fermentation. In vitro data using a commensal *Clostridium* showed outstanding [^18^F]FDG retention, validating the interaction between target GM and PET probe. Fermentation can lead to SCFA or lactic acid. The relationship linking caecum *Lactobacillaceae* and caecum [^18^F]FDG imaging signals was inverse in the pool of mice and became positive in the sole probiotic group. This duality suggests a hierarchy, in which SCFA-producing bacteria (*Clostridia* and *Bacteroidia*) are favoured glucose consumers, whereas bacteria of lactate fermentation (e.g., *Lactobacilli*) can prevail when the former are suppressed (by antibiotic in this case) and replaced by *Lactobacilli*-enriched probiotics. Glucose is a key substrate for a variety of bacterial functions, and for body organ physiology and pathophysiology. It is the central item of metabolic syndrome and diabetes. It provides energy to brain, adipose tissue and (especially ischemic) myocardium, but high glucose levels are toxic to these organs. It feeds many types of cancer influencing malignancy, proliferation, invasion, migration and chemoresistance [2–4, 30, 31]. Insulin resistance is a risk factor in all these conditions. Our results suggest that GM PET imaging can contribute to characterize and target this metabolic risk. We showed that high [^18^F]FDG uptake in caecum bacteria lowers the proportion of ingested [^18^F]FDG entering the circulation, and the exposure of myocardium and adipose tissues to glucose, predicting lower levels of circulating insulin and IL-6 and higher insulin sensitivity. GM depletion by antibiotics redirected ingested [^18^F]FDG and glucose into blood and tissues, consistent with previous observations in perfused hearts of germ-free mice, in which glucose was suggested to compensate for the lack of bacteria-derived energy sources [27]. Probiotic bacteria inoculation may sequester some [^18^F]FDG, at the same time producing alternative substrates restoring glucose demands in white fat, whereas myocardium and brown fat seemed less adaptive. These exchanges may explain the lower glycemic levels attained in the antibiotic and probiotic groups when glucose was given orally and not by intraperitoneal route.

The sorbitol analogue [^18^F]FDS has been primarily developed for imaging enterobacterial infection when administered intravenously. However, its oral administration reveals broader interactions with GM communities. This is in full agreement with most recent discoveries [32], identifying *Clostridia* and more specifically *Lacnospiraceae*, as main bacteria catabolizing sorbitol in the gut. Accordingly in this study, [^18^F]FDS retention in the caecum was primarily associated with the abundance of *Clostridia* class bacteria, including *Lacnospiraceae* and *Oscillobacter*, with a contribution of *Alistipes* and *Muribaculum* (*Bacteroidia* class). Importantly, [^18^F]FDS retention reflects bacterial uptake and intracellular trapping rather than complete sorbitol fermentation, thus serving as an in vivo marker of microbial accessibility to sorbitol substrates. Notably, certain *Alistipes* species can weakly ferment sorbitol and undergo expanded growth under sorbitol-exposure [9]. [^18^F]FDS data were weakened in multivariate and FDR statistics losing significance in adults, likely due to intra-group variability, since sorbitol is a substrate of niche (compared to glucose), and the [^18^F]FDS signal may be resilient to microbiota depletion and would likely benefit from greater dysbiosis. Sorbitol intake is related to polyol intolerance, diarrhoea, and inflammatory bowel diseases [32, 33]. Sorbitol is commonly adopted as low-calorie sweetener in foods, such as sugar-free chewing gum, candy, mints, jam, diet drinks, and chocolate [34]. In all these contexts, our oral [^18^F]FDS PET paradigm holds promise as biomarker for industrial R&D and patients’ screening.

In addition, our study supports a potential value for [^18^F]FDS PET imaging to inform on small intestinal barrier integrity, whose loss is implicated in the migration of pro-inflammatory factors from gut to body. Sorbitol can be absorbed by passive diffusion (lacking GI transporters) and is eliminated in the urine, since body tissues lack the apparatus for its use. The accumulation of [^18^F]FDS observed in bladder images (urine) was proportional to the number goblet cells in the small gut and their production of defensive mucus, which was promoted by antibiotics. The finding was specific, as goblet cells in caecum were neither affected, nor related to [^18^F]FDS excretion.

Choline is an essential nutrient involved in protection from non-alcoholic fatty liver disease, muscle damage, cognitive decline and several cancer types, though its catabolism into trimethylamine (TMA) and subsequent liver oxidation (TMAO) has been associated to higher risk of heart disease [1]. Our results support [^11^C]cho imaging as a powerful tool to probe microbiota-dependent modulation of intestinal choline handling. Despite short ^11^C half-life, the dose, scanner sensitivity and high signal to noise ratio provided sufficient image quality until late time points. We were able to visualize that the small intestine is the main site of interaction between choline and bacteria, and we observed the production of tissue radiometabolites, the main falling in the betaine, acetylcholine, TMA-TMAO TLC spectrum, and the smaller possibly including phosphatidylcholine, as based on the literature [22, 24, 35, 36]. Metabolites can be produced by GM (all, but especially acetylcholine and TMA) or liver (especially betaine, phosphatidylcholine). Supporting a GM origin, we observed that ^11^C-activity in urine (50% intact [^11^C]cho, 50% radiometabolites) was almost fully suppressed by antibiotics in vivo (bladder images), leaving more activity in the small gut; in addition, the main radiometabolite was not seen in the urine of our two antibiotic treated mice undergoing ex vivo TLC. The marked suppression of both intestinal clearance and urinary excretion of ^11^C-labelled metabolites by antibiotic treatment strongly supports a microbiota-dependent contribution to early choline metabolism, despite the acknowledged role of hepatic pathways. Prior human evidence shows that urinary excreted TMA-TMAO explain 60% of ingested choline in 24 h, but only 5% or less of excretion after 4 h of ingestion [37, 38]. In fact, our GM sequencing data were instrumental to relate the small gut washout of [^11^C]cho to the relative abundance of *Lactobacillales* and *Bacillales* genera, and *Dubosiella*, and *Enterorhabdus* genera, none of which possess the CutC/D gene cluster required to produce TMA, whereas some *Lactobacilli* can produce acetylcholine [39]. This finding does not exclude the presence of TMA forming bacteria, which are normally occurring in GM, but suggests an important role of other bacteria in clearing [C]cho from small intestine under the current study conditions. The in vitro observation of remarkable and specific interaction between [^11^C]cho and *Lactobacillales* corroborates the above reasonings.

A previous report also suggests that the urine suppression of choline metabolites by antibiotics protects against cardiovascular outcomes [38]. In our study, [^11^C]cho clearance from small gut seemed anti-inflammatory (lower IL-6 levels) but dysmetabolic (higher insulin, glucose, insulin resistance), supporting the known duality of choline metabolites regulating oxidative stress against fat-transport, absorption, accumulation and export. In all, more attention may be devoted to acetylcholine.

Our recent review of FMT studies underscores the direct and clinically significant action of GM to regulate the glucometabolic and inflammatory profile [40], which we were able to also document with the support of PET imaging in mice transplanted from donor children [25]. Here, we used mice as surrogate host to proof the principle that our imaging approach can discriminate the human GM on an individual basis, as requirement for personalized diagnostics. In fact, FMT from two age-extreme ladies was inoculated in similarly adult mice, showing very immature patterns in the infant’s GM, whereas the older lady’s GM reproduced the kinetics of [^18^F]FDG, [^18^F]FDS and [^11^C]cho seen in adult mice. Most interestingly, the multi-tracer PET approach was able to establish that the older lady’s GM would be enriched with *Clostridia* in the caecum and *Lactobacillaceae* in the small intestine, with infant’s GM *Lactobacillaceae* colonizing more dominantly the caecum. Notably, the observed imaging features strongly reflected the outcoming health profile, with protective anti-inflammatory and insulin sensitizing features from the kinetics of [^18^F]FDG, [^18^F]FDS and [^11^C]cho pertaining to the older lady’s GM. These are pilot results, and a larger portfolio of tracers is expected to characterize in more detail inter-individual differences in GM composition.

Our study has several limitations. So far, PET imaging reflects tracer handling by microbial communities and host tissues and cannot resolve single-bacterial species metabolic rates, representing a functional systems-level readout rather than a substitute for high-resolution microbial or metabolic profiling. Though our findings indicate that choline radiometabolites can become an important asset towards GM-PET based predictions, TLC timelines, dilutions and small aliquots led us to use dedicated mice after one hour of ingestion. The use of [^18^F] choline tracers may be considered as an alternative for its favourable half-life, though at the expense of dosimetry and slightly less physiological metabolism. Sample sizes were not big, but all adequate, based on power calculations and the available literature, as stimuli and the observed differences were very big and GM was not altered by different vehicles, facilitating some grouping. Groups were sex-balanced, and kinetics were visually similar in sex-subgroups, though we do not exclude that separate-sex analyses (requiring power re-evaluation) may disclose peculiarities. In all, we did not use a larger number of mice because tracer doses used in clinical practice make humans directly best suited, though exposures of GI walls need to be estimated before moving into humans with oral multi-tracer approaches. In addition, consistency with human physiology and diseases remains to be proven.

In conclusion, this study introduces a new paradigm in which GM can be characterized by PET imaging within its native operating environment, simultaneously capturing its spatial organization and its functional interaction with host health and inflammatory outcomes. By combining orally administered tracers with distinct metabolic fates, this approach enables a dynamic, in vivo assessment of dominant microbiota-associated functions along the GI tract.

The translational potential of this strategy is supported by the widespread availability of PET-CT scanners, the increasing sensitivity of next-generation systems allowing lower radiation exposure, and the extensive clinical use of [^18^F]FDG and radiolabelled choline, and prior intravenous administration of [^18^F]FDS in humans, and the GMP regulatory simplification related with oral probes. Importantly, proof-of-principle FMT experiments demonstrate that PET imaging can resolve individualized, donor-associated functionally meaningful microbiota phenotypes in vivo, even across hosts, which are not readily accessible by compositional analyses alone. Given the profound inter-individual variability of GM, this ability to functionally phenotype microbial activity at the single-subject level position PET imaging as a promising tool in precision medicine. Such an approach may enable risk stratification, prognostic assessment, and the development and monitoring of personalized microbiota-targeted interventions, including dietary modulation, pre-, pro- and post-biotics, and microbiota-directed therapeutics.

## Supplementary Information

Below is the link to the electronic supplementary material.

Supplementary Material 1

## Data Availability

The datasets generated during and/or analysed during the current study are available from the corresponding author on reasonable request.
