# Supplementary material for: Functional PET imaging of gut microbiota with [18F]fluorodeoxyglucose, [18F]fluorodeoxysorbitol and [11C]choline reflects Clostridia and Lactobacillales abundance in caecum and small intestine and host metabolic interactions
Source: Eur J Nucl Med Mol Imaging. 2026 Jun 13;53(10):6111–27. doi: 10.1007/s00259-026-07988-y (PMC13421236; doi:10.1007/s00259-026-07988-y)
Supplement: Supplementary file 1 — Supplementary Material 1 [file 259_2026_7988_MOESM1_ESM.pdf]

Supplementary Information

Functional PET imaging of gut microbiota with [^{18}F]fluorodeoxyglucose, [^{18}F]fluorodeoxysorbitol and [^{11}C]choline reflects *Clostridia* and *Lactobacillales* abundance in caecum and small intestine and host metabolic interactions

Federica La Rosa, Maria Angela Guzzardi, Gabriele Conti, Debora Petroni, Mercedes Pardo Tendero, Silvia Bernardi, Monica Barone, Daniele Panetta, Lorena Tedeschi, Costanza Fabbri, Federico Casavecchia, Daria Riabitch, Federico Granziera, Rosetta Ragusa, Chiara Caselli, Assuero Giorgetti, Daniela Campani, Emma Baglini, Luca Menichetti, Philip Elsinga, Gert Luurtsema, Patrizia Brigidi, Patricia Iozzo

Corresponding author:

Patricia Iozzo, MD, PhD, Institute of Clinical Physiology, National Research Council (CNR), Via G. Moruzzi 1, 56124, Pisa, Italy. Email: patricia.iozzo@cnr.it

Supplementary Table 1. Post-hoc testing for repeated measures across study groups in the emerging intestinal segments (caecum and small gut), from data shown in Figures 2-5.

	Fisher's LSD Bonferroni/Dunn	Scheffé's test	Tukey/Kramer Student-Newman-Keuls
[^{18}F]FDG adults			
Untreated vs antibiotic*^	<0.0001	<0.0001	*^
Untreated vs probiotic*^	<0.0001	0.0003	*^
Antibiotic vs probiotic	0,4162	0,7176	0,7176
[^{18}F]FDG young			
Untreated vs antibiotic*^	0.0417	0.0417	*^
[^{18}F]FDS adults			
Untreated vs antibiotic	0.0209	0.0684	
Untreated vs probiotic	0.0892	0.2341	
Antibiotic vs probiotic	0.6920	0.9241	
[^{18}F]FDS young			
Untreated vs antibiotic*^	0.0417	0.0417	*^
[^{11}C]cho adults			
Untreated vs antibiotic*^	<0.0001	0.0002	*^
Untreated vs probiotic*^	0.0040	0.0158	*^
Antibiotic vs probiotic	0.4178	0.7193	

Measures include all imaging time points from 1h. In post-hoc analyses, significance according to the first two tests is <0.05 and 0.0167, respectively. Symbols in the last column (*^) indicate significance also in *Tukey/Kramer and ^Student-Newman-Keuls tests. Young [^{11}C]cho mice were not tested due to the absence of significance between groups already in single point comparisons in Figure 5.

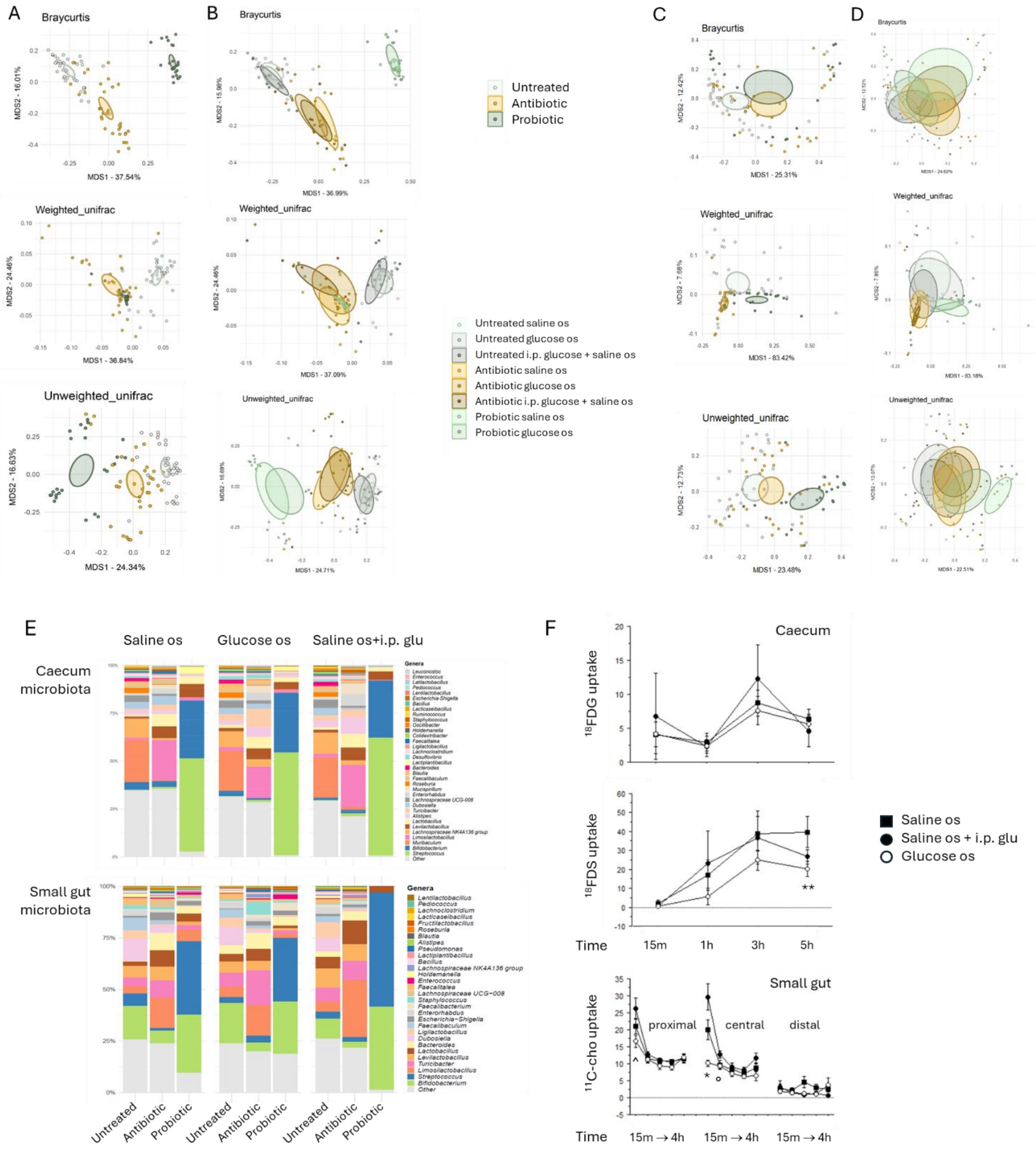

G	small intestine						caecum					
	Unweighted		Weighted		Bray-Curtis		Unweighted		Weighted		Bray-Curtis	
	p	p.adj	p	p.adj	p	p.adj	p	p.adj	p	p.adj	p	p.adj
Untreated vs antibiotic	0,1102	0,1102	0,0010	0,0015	0,0002	0,0003	0,0000	0,0000	0,0000	0,0000	0,0000	0,0000
Antibiotic vs probiotic	0,0008	0,0012	0,0385	0,0385	0,0001	0,0003	0,0000	0,0000	0,0000	0,0000	0,0000	0,0000
Untreated vs probiotic	0,0001	0,0003	0,0003	0,0009	0,0053	0,0053	0,0000	0,0000	0,0000	0,0000	0,0000	0,0000
Antibiotioc saline os + i.p. glu vs saline os	0,87	0,88	0,23	0,31	0,79	0,83	0,95	0,95	0,21	0,26	0,31	0,37
Antibiotic saline os + i.p. glu vs glucose os	0,88	0,88	0,58	0,62	0,45	0,53	0,85	0,92	0,60	0,70	0,78	0,88
Untreated saline os vs glucose os	0,55	0,70	0,96	0,96	0,80	0,83	0,79	0,92	0,80	0,87	0,96	0,96
Untreated saline os + i.p. glu vs glucose os	0,46	0,66	0,45	0,55	0,90	0,90	0,86	0,92	0,66	0,74	0,95	0,96
Untreated saline os + i.p. vs saline os	0,47	0,66	0,32	0,41	0,64	0,71	0,90	0,94	0,90	0,90	0,85	0,92
Antibiotic saline os vs glucose os	0,59	0,72	0,49	0,57	0,41	0,50	0,80	0,92	0,60	0,70	0,39	0,45
Probiotic saline os vs glucose os	0,03	0,09	0,94	0,96	0,08	0,14	0,16	0,20	0,85	0,88	0,23	0,30

S1. Typical distance metrics show differences between treatments in caecum and small gut (A, C), including presence/absence (unweighted) and relative abundance of taxa (weighted). Significance levels for a-e are given in the bottom table (G). Within each treatment, the vehicle type did not interfere with β-diversity (B, D) or composition (E), whose significance levels are reported in G; functionally (F), bacteria uptake of [^{18}F]FDS was reduced by the glucose vehicle, not affecting [^{18}F]FDG retention ($p>0.40$) or [^{11}C]cho kinetics at relevant times (2-3 hours); **$p=0.01$ oral glucose vs saline; *$p<0.05$ vs all groups; ^$p<0.05$ oral vs i.p. glucose; °$p<0.05$ i.p. glucose vs saline.

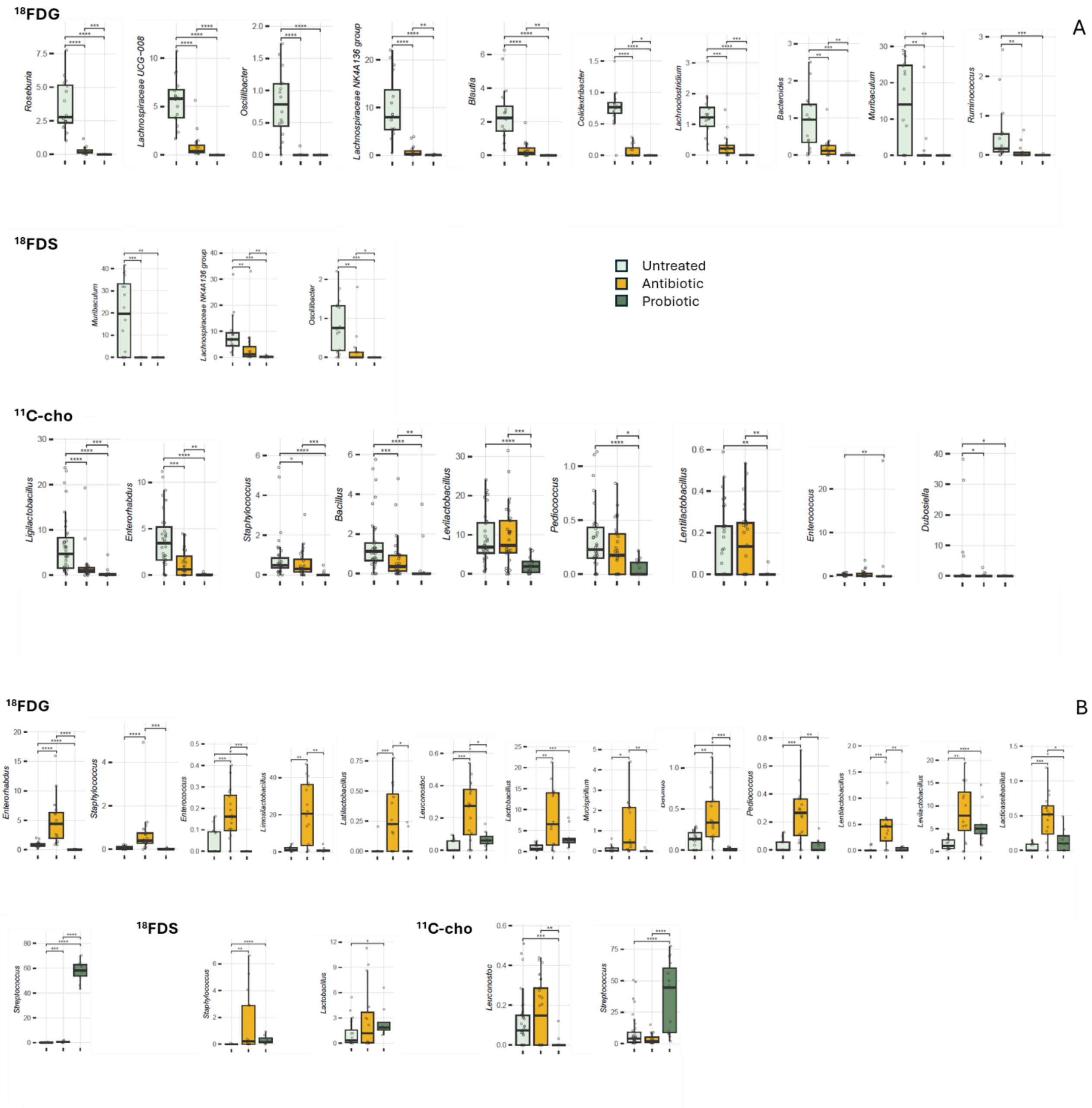

S2. Bacteria taxa identified in regression analysis in Figure 6 are shown in the treatment groups. Panel A includes bacteria that were proportional to the PET imaging signal, namely underlying higher retention of ^{18}F tracers in caecum and higher clearance (lower values) of [^{11}C]cho in small proximal-central intestine. Panel B indicates inversely related bacteria, whose absence may reduce retention of ^{18}F tracers or may reduce [^{11}C]cho clearance, augmenting its permanence in the small intestine. Respective FDR corrected statistics are given in S3.

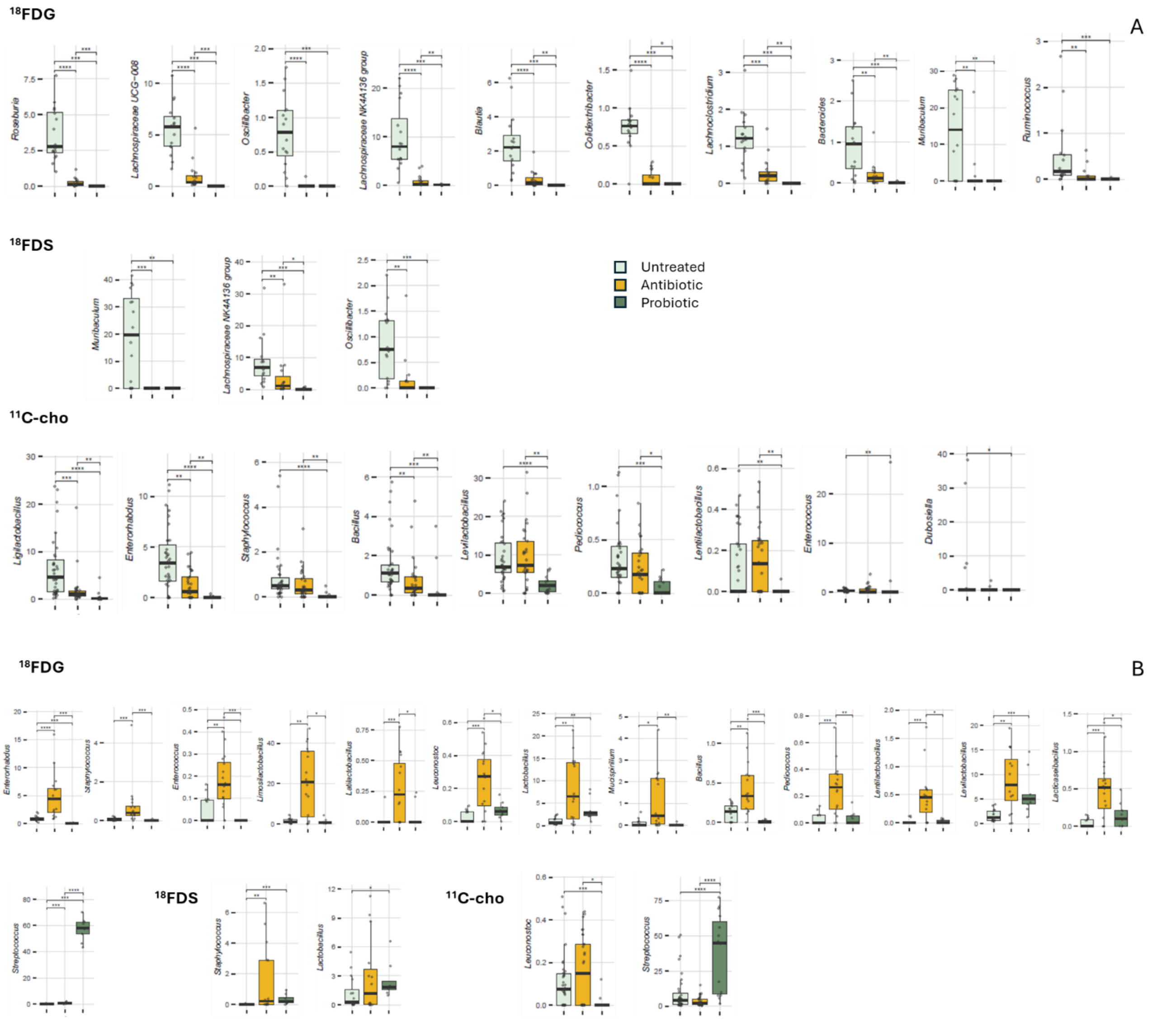

S3. FDR corrected analysis of the bacteria taxa shown in S2, with the same AB panel labelling, demonstrating that high statistical significance is maintained with more stringent statistics.

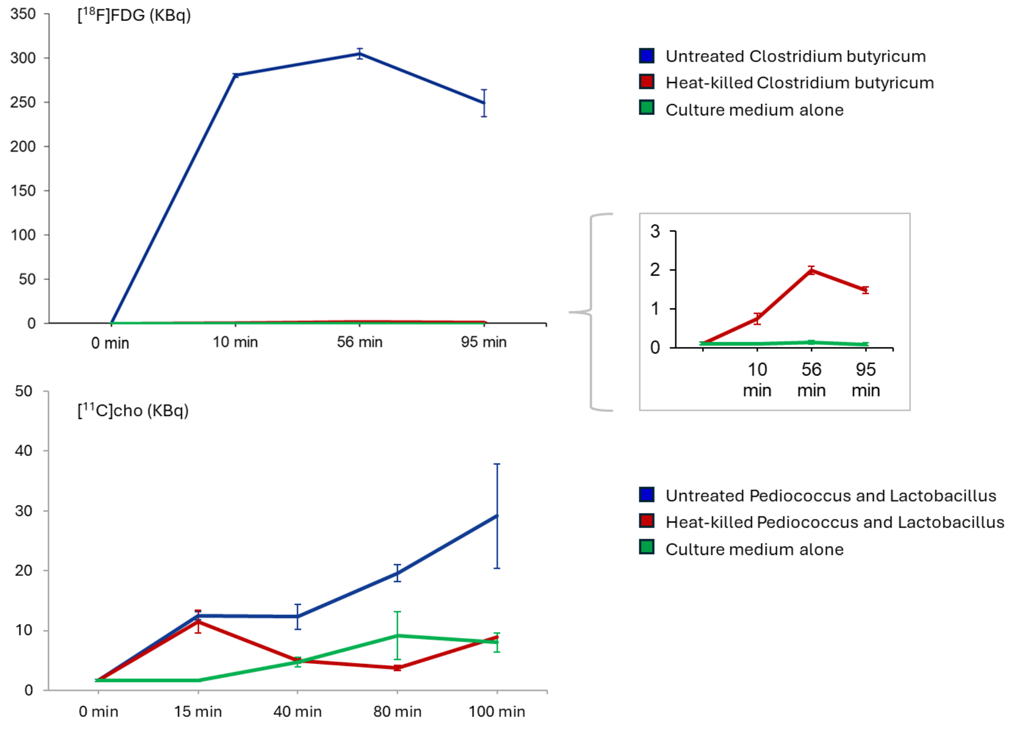

S4. Bacteria were cultured from commercial probiotics, selected on the taxonomic families emerging in this study, namely *Clostridiaceae* (*Clostridium butyricum* for [^{18}F]FDG) and *Lactobacillaceae* (*Pediococcus acidilactici* and *Lactobacillus plantarum* species for [^{11}C]cho). Bacteria were separately cultured overnight in degassed Luria Broth at 37 °C, and colony forming units (CFU) were determined by plating aliquots in serial dilutions on Luria Broth-Agar plates for overnight incubation at 37°C in low-oxygen atmosphere. *Clostridia* (3x10^6 CFU) were exposed to [^{18}F]FDG, and *Lactobacillaceae* (2x10^8 CFU) were exposed to [^{11}C]cho. Tracers were added to working aliquots of untreated bacteria or heat-killed (99°C for 5 min) bacteria or Luria Broth not containing bacteria (negative control). The three conditions were examined in duplicate samples. Each vial was exposed to 0.39 MBq of [^{18}F]FDG or 5.4 MBq of [^{11}C]cho. Incubation was carried out at 37 °C on a static thermoblock. Samples were collected from each vial at given time points for 90-100 minutes and washed five times with sterile phosphate-buffered saline (PBS) by centrifugation (4 minutes, 4000 rpm). Then, radioactivity in bacterial pellets counted, and decay corrected.

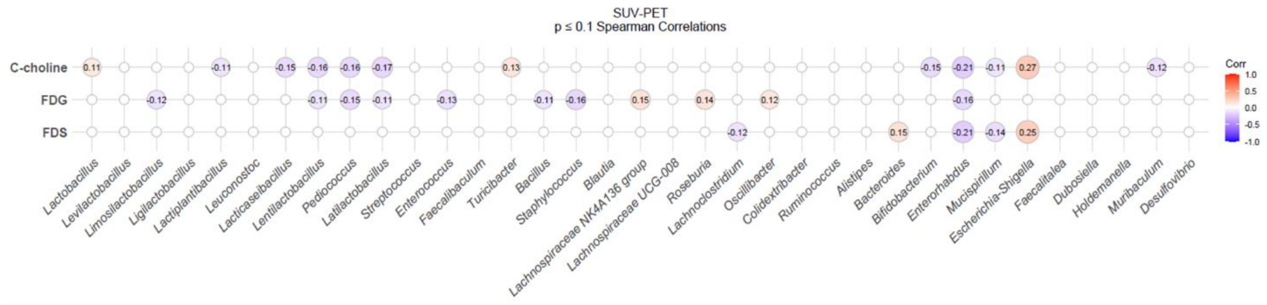

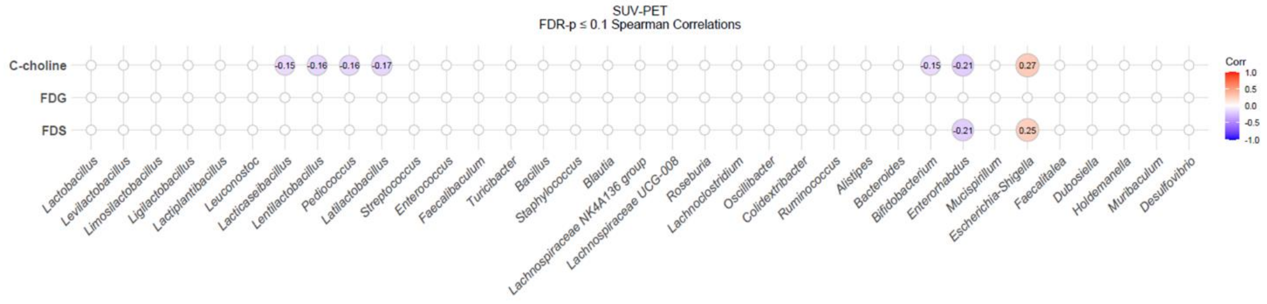

S5. Regression analyses performed between PET signals in all GI tracts and corresponding microbiota composition highlight the expected uptake of ^{18}F tracers by Enterobacteria, as shown by positive correlations with Escherichia-Shigella genera in univariate analysis (top panel), whose strength and significance survived FDR correction (bottom panel).

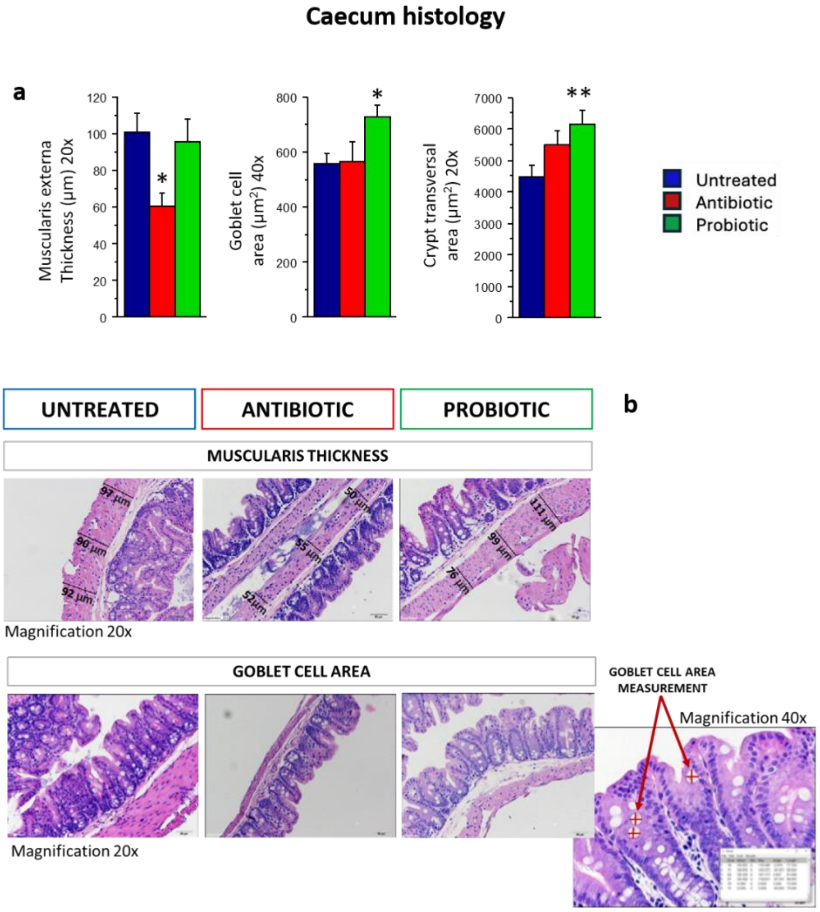

S6. Caecum histology in the treatment groups, showing (a) reduced muscular thickness in the antibiotic treated vs other groups (*p<0.05), and expanded goblet cell area and number in the probiotic vs the untreated group (**p<0.01). The bottom panel (b) provides representative examples of hematoxylin-eosin-stained slices of caecum in the given groups.

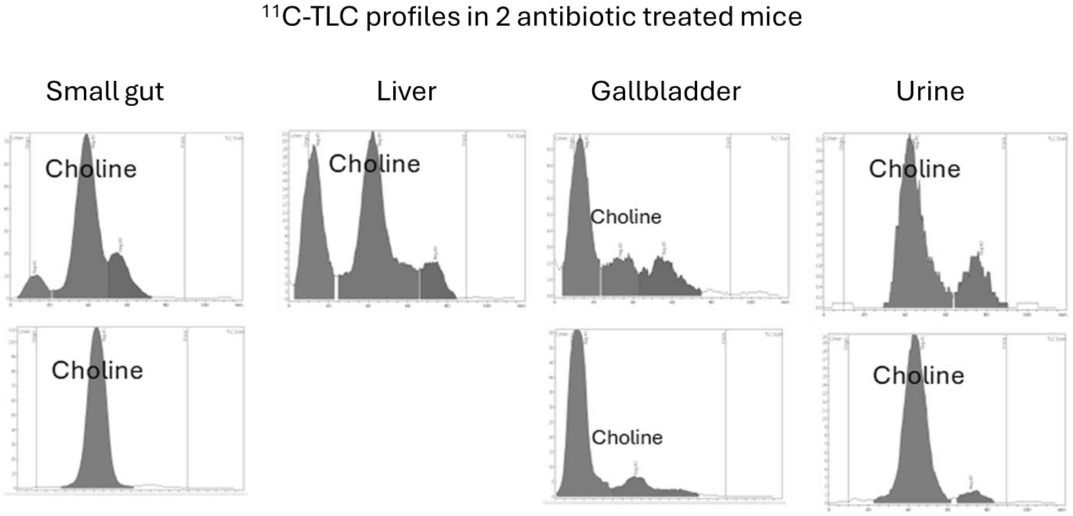

S7. Explorative assessment of tissue ^{11}C-activity in two antibiotic treated mice identifies maximally three peaks, as in the untreated case of Figure 4, but slower hepatic formation and absent urinary excretion of the major choline radiometabolite compared with Figure 4.
